# Use what you can: storage, abstraction processes, and perceptual adjustments help listeners recognize reduced forms

**DOI:** 10.3389/fpsyg.2014.00437

**Published:** 2014-05-30

**Authors:** Katja Poellmann, Holger Mitterer, James M. McQueen

**Affiliations:** ^1^Language Comprehension Department, Max Planck Institute for PsycholinguisticsNijmegen, Netherlands; ^2^International Max Planck Research School for Language SciencesNijmegen, Netherlands; ^3^Behavioural Science Institute and Donders Institute for Brain, Cognition and Behaviour, Radboud University NijmegenNijmegen, Netherlands

**Keywords:** reduction, word-specificity, generalization, learning, adaptation, eye-tracking

## Abstract

Three eye-tracking experiments tested whether native listeners recognized reduced Dutch words better after having heard the same reduced words, or different reduced words of the same reduction type and whether familiarization with one reduction type helps listeners to deal with another reduction type. In the exposure phase, a segmental reduction group was exposed to /b/-reductions (e.g., *minderij* instead of *binderij*, “book binder”) and a syllabic reduction group was exposed to full-vowel deletions (e.g., *p'raat* instead of *paraat*, “ready”), while a control group did not hear any reductions. In the test phase, all three groups heard the same speaker producing reduced-/b/ and deleted-vowel words that were either repeated (Experiments 1 and 2) or new (Experiment 3), but that now appeared as targets in semantically neutral sentences. Word-specific learning effects were found for vowel-deletions but not for /b/-reductions. Generalization of learning to new words of the same reduction type occurred only if the exposure words showed a phonologically consistent reduction pattern (/b/-reductions). In contrast, generalization of learning to words of another reduction type occurred only if the exposure words showed a phonologically inconsistent reduction pattern (the vowel deletions; learning about them generalized to recognition of the /b/-reductions). In order to deal with reductions, listeners thus use various means. They store reduced variants (e.g., for the inconsistent vowel-deleted words) and they abstract over incoming information to build up and apply mapping rules (e.g., for the consistent /b/-reductions). Experience with inconsistent pronunciations leads to greater perceptual flexibility in dealing with other forms of reduction uttered by the same speaker than experience with consistent pronunciations.

## Introduction

In casual speech, speakers tend to articulate in a sloppy way. They frequently reduce words by slurring and even omitting segments or syllables (Ernestus, 2000; Patterson et al., 2003; Johnson, 2004; Mitterer and McQueen, 2009). A given native Dutch speaker may for example reduce the /b/ in *bandiet* “bandit” to [m] or leave out the first vowel in *kanaal* “canal” (Schuppler et al., 2011). Listeners might get used to such pronunciation habits; they may recognize a reduced word better the second time and they may be able to adjust rapidly to new forms of reduction produced by the same speaker. The present study investigates *whether* listeners adapt to a given reduction type (/b/-reductions or full-vowel-deletions) and, if so, *how* they adapt by asking if they can apply their knowledge to previously unheard reduced words of the same reduction type and/or of the other reduction type. Put another way, the present study tests word-specific learning effects as well as generalization of learning within and across reduction types.

Listeners are usually not aware that they encounter numerous reduced word forms every day (Kemps et al., 2004; Ernestus and Warner, 2011). They use the information provided by the sentence context or also the wider discourse context to predict and, if necessary, restore the upcoming word (Ernestus et al., 2002; Brouwer et al., 2013). On a lower level, listeners are also able to exploit the fine phonetic detail present in reduced forms to distinguish for instance between a reduced form [spɔːt] of *support* and the unreduced form [spɔːt] *sport* (Manuel, 1992).

Another mechanism which listeners may use to recognize reduced forms better is adaptation, as perceptual learning may be especially important when the conditions for spoken-word recognition become challenging.

Adaptation, for instance, has been found to play a crucial role in recognizing regional and foreign-accented speech (Clarke and Garrett, 2004; Floccia et al., 2006; Mitterer and McQueen, 2009). Listeners are able to adapt rapidly to these deviant pronunciations and can apply their acquired knowledge to the way they process other words (Witteman et al., 2013).

The present study tests whether a similar adaptation process also takes place when listeners encounter reduced words in their native language. Like regional and foreign-accented words, reduced words are also variants of canonical pronunciations, but the reduction types chosen for investigation in the present study (/b/-reductions and full-vowel-deletions) were not regionally marked. In contrast to regional and foreign accents, reductions affect predominantly unstressed segments and syllables. They are therefore probably less salient. This might make it harder for listeners to adapt to reduced speech than to regional or foreign-accented speech.

The present study investigates potential adaptation processes and their possible constraints. Consider a Dutch listener hearing the word *paraat* “ready” pronounced as *p'raat*. Different patterns of adaptation are possible that vary in how general they are. First, no adaptation whatsoever may be found. Second, the listener may find it easier to recognize a second instance of the same word with the same reduction pattern. This would be similar to the recognition benefits for words repeated in the same voice that provide some of the evidence for episodic models of word recognition (Nygaard et al., 1994; Goldinger, 1996, 1998; Nygaard and Pisoni, 1998). Third, listeners may learn that this speaker generally deletes vowels in unstressed syllables. This abstractionist learning may be quite specific, so that only very similar reductions to *p'raat* benefit (e.g., *Parijs* “Paris” produced as *P'rijs*; note that the Dutch rendition is stressed on the second syllable) or it may include reductions of unstressed vowels in other contexts (e.g., *kanaal* “canal” produced as *k'naal*). The strongest possible generalization would be that the listener assumes that this speaker reduces a great deal and hence finds it easier to recognize any kind of reduction uttered by the speaker.

Finding a word-specific learning effect, that is, better recognition of a reduced word on hearing it for the second time compared to the first time, would be evidence for episodic storage of reduced forms. In contrast, observing generalization of learning to new words of the same reduction type (e.g., generalization from *p'raat* to *P'rijs* or *k'naal*) would indicate that an abstraction process is taking place and that it occurs at a prelexical level. Storing reduced forms alone cannot account for easier recognition of previously unheard reduced words (McQueen et al., 2006; Cutler et al., 2010). In a purely episodic account of lexical access, there is no way to adjust weights of sublexical units like segments and syllables to build up rules that capture regular reduction processes (e.g., “*Potentially restore a bilabial nasal in an unstressed syllable to a bilabial voiced stop if followed closely by another nasal*”). Finding generalization of learning to new words of the same reduction type would thus support the claim that there is abstraction in lexical access. Observing generalization of learning from one reduction type to another may also be evidence for abstraction—if there is enough similarity between the reduction types to abstract over the respective mapping rules. Consider, for example, two types of prefix reductions, such as *ge*- /gə/ → /g/ and *be*- /bə/ → /b/ in German. An abstraction rule may be: “*Potentially insert a schwa after an initial voiced stop*” (instead of “… *after an initial voiced velar/bilabial stop*”). However, should generalization of learning across reduction types be found for very different reduction types, such as the /b/-reductions and full-vowel-deletions examined here, this would more likely indicate a non-specific adjustment and be evidence for the flexibility of the perceptual system. That is, instead of specific adaptation processes (storage of reduced forms and/or abstraction of reduction rules), listeners could make a more general adjustment to the current talker's speaking style.

To test these possible adaptation effects, the printed-word eye-tracking paradigm (McQueen and Viebahn, 2007) was used. In the exposure phase, one group of participants was exposed to segmental reductions, another group was exposed to syllabic reductions and a third group was exposed only to canonical pronunciations. The first group, the segmental reduction group, heard /b/-reductions, where the word-initial /b/ was reduced to a bilabial nasal (e.g., *minderij* instead of *binderij* “book binder”). The second group, the syllabic reduction group, heard words in which the first, unstressed full vowel was deleted (e.g., *p'raat* instead of *paraat* “ready”). The third group, the control group, heard the same words as the two experimental groups during the exposure phase but all in unreduced form (e.g., *binderij* and *paraat*).

In order to assess the frequency with which our chosen reduction types (/b/-reductions and full-vowel-deletions) occur in spontaneous speech, we conducted a corpus study following the principles of Pluymaekers et al. (2005). First, all sound files containing a /b/-initial word with a nasal in third position and an unstressed first syllable were extracted from the Corpus of Spoken Dutch (Oostdijk, 2000). Per word type (this notion here not only describes words belonging to different lemmas but also different word forms of one lemma, e.g., an inflected verb form or the plural of a noun) only one token was randomly chosen to determine its phonetic realization. Out of 65 word types, six showed a /b/ → [m] reduction in the first segment (i.e., 9.2% of the considered cases). A similar analysis was conducted to assess the frequency of full-vowel-deletions in initially unstressed words. The vowel was deleted in eight out of 66 word types (i.e., in 12.1%) containing either a voiceless plosive (/p/, /k/) or a voiceless velar fricative (/x/) in first position and an alveolar nasal or liquid in third position. This was also the segmental structure used in the syllabic reduction condition. The chosen reduction types were thus indeed real-world phenomena and comparable in terms of frequency.

These two reduction types were chosen to examine adaptation to two different-sized linguistic units, the phoneme and the syllable, and the possible interaction of the adaptation effects. An earlier study showed that listeners adapt to syllabic reductions involving a morpheme: After exposure to words containing the reduced prefix *ver*- (realized as [fː]), Dutch listeners recognized previously unheard reduced *ver*-words better than a control group (Poellmann et al., under revision). In the present study, we test whether this is also the case for non-morphemic syllables. The deletion of the unstressed, full vowel in CVC-initial words like *paraat* always leads to a reduction in the number of syllables, which is why this reduction type is called “syllabic.” A pure comparison of morphemic and non-morphemic reductions, however, turned out to be impossible in Dutch. Ideally, one would like to compare a morphemic reduction type (that only affects one specific morpheme, i.e., the same strings of segments, such as Dutch *ge*-) to a non-morphemic reduction type that also only affects one specific string of segments (e.g., *pa*-). The Dutch lexicon, however, does not contain enough words starting with one specific unstressed non-morphemic syllable to conduct such an experiment. This constraint on the (non-)morphemic status hence leads inevitably to higher variability in the segmental structure of the CVC-targets compared to the *ver*-targets examined in Poellmann et al. (under revision). This difference in the degree of consistency with which words are reduced in the two conditions allowed us to ask whether phonological consistency determines which adaptation processes (e.g., storage, abstraction rules, general flexibility) listeners are able to use.

In the test phase, all three groups of participants heard /b/-reductions and vowel-deletions. The reduced words were either the same as in the exposure phase (in Experiments 1 and 2) or different (in Experiment 3). If listeners adapt to a given reduction type and if they can transfer this knowledge to new words (Experiment 3) and/or to other reduction types (Experiments 1–3), participants in the experimental groups should recognize reduced words better than participants in the control group.

Regardless of the specifics concerning the reduction (such as size of the reduced unit or input consistency), it seems plausible that a reduced word can be recognized more easily if it is encountered a second time. We therefore expect to find word-specific learning effects for both /b/-reductions and vowel-deletions.

Moreover, we predict that learning about /b/-reductions generalizes to new words that are reduced in the same way. Such generalization effects have been observed for a similar kind of /b/-reduction where the word-initial voiced stop was reduced to a labio-dental approximant [ν] (Poellmann et al., under revision) and for learning about segmental idiosyncrasies (McQueen et al., 2006). In the McQueen et al. (2006) study, listeners adapted to an ambiguous sound (between /s/ and /f/) and transferred their knowledge to previously unheard minimal pairs that only differed in containing either /s/ or /f/.

The predictions concerning within-reduction-type generalizations for full-vowel-deletions are less clear. The constraint on the (non-)morphemic status of the syllable leads to higher variability in the segmental structure of the CVC-targets compared to the /b/-targets. If the input has to be highly consistent for the creation of abstract mapping rules, we might not observe generalization of learning.

The two reduction types under investigation differ in several respects, such as the degree of reduction (weakening of the [b] vs. deletion of the vowel), in the segment that is reduced (bilabial voiced stop vs. full vowel) and in the position the reduced segment occurs (first position for /b/-reductions vs. second position for vowel-deletions). In order to observe generalization of learning across reduction types, listeners would hence have to adapt on a fairly global level. However, such global adjustments to challenging listening conditions have been observed before (Brouwer et al., 2012; McQueen and Huettig, 2012).

## Experiment 1

The aim of Experiment 1 was to test whether listeners are able to recognize segmental and syllabic reductions better when they have already encountered the same words in reduced form before. Experiment 1 also asked whether learning about reductions might generalize from one reduction type to another (i.e., from /b/-reductions to full-vowel deletions and/or vice versa). In the exposure phase, one group was exposed to /b/-reductions (segmental reduction group), a second group was exposed to full-vowel deletions (syllabic reduction group), while a third group was exposed to canonical forms only (control group). In the test phase, all three groups were tested on reduced-/b/ words and vowel-deleted words. Importantly, these reduced words had already occurred in reduced or canonical form (depending on the group) in the exposure phase. If listeners can adapt to reduced words, the segmental reduction group should recognize the reduced-/b/ words better than the syllabic reduction group and the control group because of their previous exposure to these words in reduced form. The same holds for participants in the syllabic reduction group: If they can adapt to vowel-deleted words, they should perform better on these words than the segmental reduction group and the control group. If listeners can additionally transfer their knowledge about one reduction type to another, the segmental reduction group should outperform the control group on the vowel-deleted words and the syllabic reduction group should outperform the control group on the reduced-/b/ words.

### Methods

#### Participants

Seventy-five participants of the Max Planck Institute's subject pool, all native speakers of Dutch, were paid to take part. All reported normal hearing and normal or corrected-to-normal vision.

#### Design

Participants were randomly assigned to one of three groups: a segmental reduction group, a syllabic reduction group and a control group. They listened to sentences, saw four printed words on a computer screen and were asked to click on the word that occurred in the sentence. Improved word recognition in a visual-world eye-tracking experiment can be reflected by faster and more accurate mouse clicks on the target word as well as higher fixation proportions toward the target and away from the similar sounding competitor. We thus measured Reaction Times (RTs) and accuracy of mouse clicks and fixation behavior.

In the exposure phase, participants were exposed to words that were potentially reduced (see the experimental exposure trials in Table 1) but which did not appear on the screen. Instead, they saw (and had to click on) target words that occurred later in the sentences. All three groups were also exposed to unreduced /m/- and unreduced consonant-cluster-words (e.g., /mɑtros/ *matroos* “sailor” and /knɔflok/ *knoflook* “garlic”); they also had to click on these filler stimuli.

**Table 1:** **Experimental design and types of stimuli in Experiments 1 and 2**.

	**Trial type**	**Canonical word-form**	**Segmental reduction group**	**Syllabic reduction group**	**Control group**
			**/b/ → [m]**	**Full vowel deletion**	**No reduction**
Exposure phase	Experimental	/bɪndərεɪ/	[**m**ɪndərεɪ]	[bɪndərεɪ]	[bɪndərεɪ]
	Filler	/mɑtros/	[mɑtros]	[mɑtros]	[mɑtros]
	Experimental	/parat/	[parat]	[**pr**at]	[parat]
	Filler	/knɔflok/	[knɔflok]	[knɔflok]	[knɔflok]
Test phase	Experimental	/bɪndərεɪ/		[**m**ɪndərεɪ]	
	Filler	/murɑs/		[murɑs]	
	Experimental	/parat/		[**pr**at]	
	Filler	/xlɑns/		[xlɑns]	

*Reduced segments are marked bold. The potentially reduced /b/-initial words and the vowel-deleted words of the exposure phase were repeated in reduced form in the test phase*.

In the test phase, all three groups heard reduced /b/-words and vowel-deleted words in the experimental trials. These were the same words as had appeared in the exposure phase (e.g., [mɪndərεɪ] instead of [bɪndərεɪ] *binderij* “book binder” and [prat] instead of [parat] *paraat* “ready”). All groups also heard new canonical /m/- and new canonical consonant-cluster words. The reduced /b/-words, the vowel-deleted words, the unreduced /m/-word fillers and the consonant-cluster filler words were all targets and were therefore displayed on the computer screen in (canonical) orthographic form.

#### Materials

**Figure 1:**
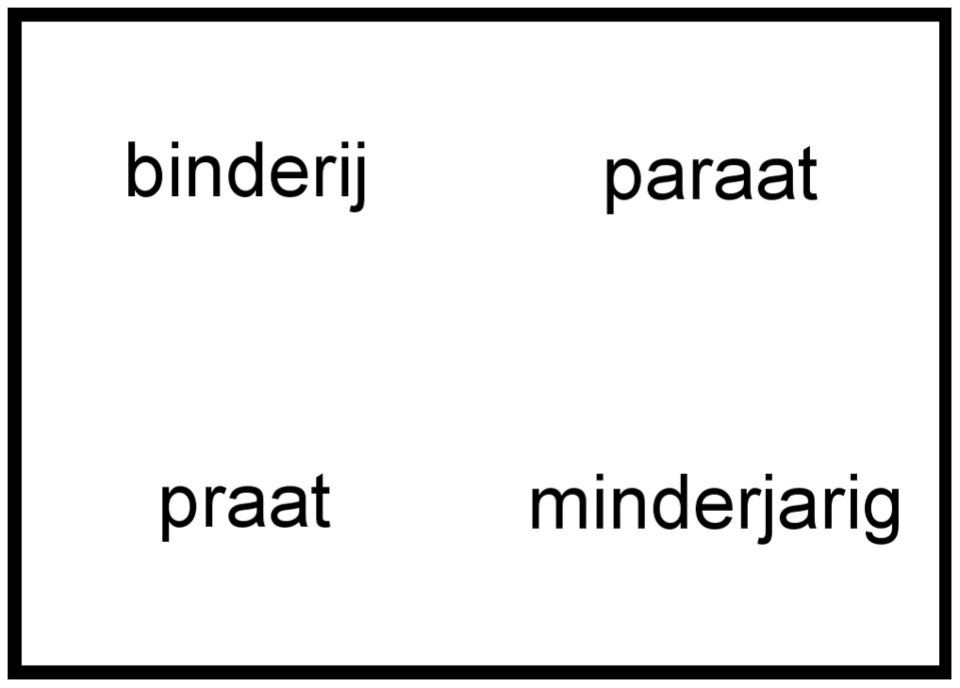
**Example display of a test trial in Experiments 1–3**.

The target words (i.e., the words participants had to click on) appeared toward the end of spoken sentences. Each target word occurred in a different sentence context not containing any further /b/s in unstressed syllables or any further unstressed CVC-sequences which would result in legal consonant clusters when omitting the vowel. The potentially reduced item occurred before the target word in the experimental trials (e.g., *Pas in een [b]/[m]inderij wordt een boek of **tijdschrift** afgemaakt* “Only at a book binder, a book or **magazine** gets finished,” where bold font indicates the target word and underlining marks the potentially reduced critical item). This was done to prevent participants from clicking on the same words twice, once in the exposure phase and once in the test phase. In the test phase, the semantic contexts preceding the target words were kept uninformative (e.g., *Het tekstverwerkingprogramma kende het woordje **[m]inderij*** niet “The word processor did not know the word **book binder**”). During each sentence, there were always four printed words on the screen. In the test trials, these were a /b/-word, a /m/-word, a CVC-word and a consonant-cluster word (see Figure 1 for an example display).

The test phase consisted of 48 experimental trials containing either /b/-targets or CVC-targets and 48 filler trials containing either /m/-targets or CC-targets. For each type of target word (/b/-target, /m/-target, CVC-target, and consonant-cluster-target), 24 target-competitor pairs were selected (see Table S1 for the /b/-targets, the CVC-targets, and their respective competitors). If a /b/-word was the target, a /m/-word was the competitor and vice versa. The same holds for CVC- and consonant-cluster-targets. All /b/- and /m/-initial words contained an unstressed first syllable. In second position, any vowel including schwa could occur followed by a nasal in third position. The latter condition was necessary for all /b/-targets to motivate nasalization at the beginning of the word. However, there are not sufficient /m/-initial words in Dutch containing a nasal in third position to create perfectly matched pairs of /b/-targets and /m/-competitors. Ideally, /b/-words and /m/-words should be as similar as possible with as much overlap in the reduced forms as possible (e.g., *binderij* “book binder” pronounced as [mɪndərεɪ] overlaps in the first two syllables with [mɪndərjarəx] *minderjarig* “underage”). Due to the infrequent occurrence of a nasal in third position following an /m/ in first position, the /m/-targets contained a random consonant in third position (and so did the corresponding /b/-competitors; e.g., *moeras* “swamp” and *boerin* “farmer's wife”). Target-competitor pairs were further matched in terms of number of syllables, stress pattern and word frequency [taken from SUBTLEX-NL (Keuleers et al., 2010)] as much as possible (see Table S1).

The principles of as much overlap and similarity as possible between targets and competitors also applied to the (reduced) CVC- and (unreduced) consonant-cluster-words. CVC-words started with an open syllable, consisting of a voiceless consonant (either /p/, /k/, or /x/) and a full vowel, followed by a liquid or /n/ in third position (e.g., *paraat* “ready”), so that the sequence resulting from vowel deletion would be a phonotactically legal consonant cluster in Dutch. The consonant-cluster words started with the same voiceless consonants directly followed by a liquid or [n] (e.g., *praat* “talk”). While the stress of the CVC-words was on the second syllable, the consonant-cluster-words were stressed on the first syllable, so that both word types were matched on stress pattern when the full vowel of the CVC-words was deleted (e.g., p'RAAT for paRAAT “ready” and PRAAT “talk”). Again, target-competitor pairs were matched on number of syllables (in the reduced form) and word frequency (see Table S1).

The exposure phase consisted of 96 trials in total. Half of them were filler trials containing /m/-targets or CC-targets. The 48 experimental trials contained potentially reduced /b/-words or CVC-words that did not appear on the screen. The only constraint for the target-“competitor” pairs on the screen was that they did not overlap.

#### Stimulus construction

Digital recordings of the stimuli were made by a female native speaker of Dutch in a sound-proof booth, sampling at 44.1 kHz. She was instructed to produce the sentences in a casual way, not just reading them aloud. For sentences containing canonically pronounced /b/-targets, an additional set containing reduced forms was created by replacing the /b/ with an /m/ from a word with the same vowel context. The spliced parts were adjusted in pitch (with PSOLA in PRAAT, Boersma and Weenink, 2010) and intensity to their new context. The transitions in amplitude preceding and following the spliced-in [m]s were smoothed where necessary in order to reduce splicing artifacts. The set of sentences containing reduced CVC-words was created by cutting out the first (unstressed) vowel of the recorded versions of these words with intact vowels. Sentence contexts were thus identical across the reduced and unreduced forms of each target word. Filler sentences containing /m/- and consonant-cluster-targets were not manipulated.

#### Procedure

Participants were seated in a sound-attenuated booth at a comfortable viewing distance from the computer screen. Eye movements were monitored using an SR Research EyeLink 1000 set-up, sampling at 1 kHz. The auditory stimuli were presented to the participants over headphones. Prior to the experiment, participants received written instructions that informed them that they would see four printed words on the screen and asked them to click on the word that occurred in the sentence.

At the beginning of each trial, a fixation cross appeared in the center of the screen for 500 ms. Four printed words (in a 25-point Arial font) were then presented. After 1500 ms, the auditory stimulus was played. As soon as participants had listened to the entire sentence and had clicked with the mouse on the screen, the following trial was initiated. Every 10 trials, a drift correction was carried out. Participants had the opportunity to take a break after every 50th stimulus. The experiment started with six practice trials. The 96 exposure trials in random order were followed by 96 test trials in random order. Randomization was different for each participant. An experimental session took approximately 25 min.

### Results

#### Exclusion criteria

Mouse click responses (reaction time and accuracy data) and eye movements served as dependent variables. For the eye-tracking data, we analyzed the data from the participant's right eye. For the analysis of the eye-tracking data, a total of 2.9% of the trials were excluded, because participants either appeared to have looked away from the screen (2.0%) or failed to click on the target or the potentially confusable competitor (0.9%). Clicks on the competitor were not excluded from all of the analyses, as the competitors sometimes better fitted the exact auditory input with reduced forms than the targets. For instance, reduced *p'raat* better fitted the canonical form of the competitor *praat* than the canonical form of the target *paraat*. Furthermore, the semantics of the test sentences did not make clear which word was the target. In the case of minimal pairs such as *paraat* and *praat*, participants thus never received disambiguating information about which of the two words they should click on. Therefore, clicks on competitors were not regarded as errors in the analyses of the eye-tracking and the reaction time data. Note also that excluding trials from the eye-tracking analysis in which participants clicked on the competitor would invalidate any learning effects. Presumably, participants look more at the competitor when they click on it. Excluding these trials would result in a greater preference for the target over the competitor and would thus misleadingly indicate a greater learning effect than was actually present. Moreover, the focus in the RT analyses is on the comparisons across the three exposure groups; these comparisons are thus orthogonal to any differences between targets and competitors. Click responses to competitors, however, were regarded as incorrect in the analysis of the accuracy scores.

The upper part of Table 2 displays descriptive statistics on RTs for trials in which participants clicked either on the target or on the phonological competitor in the test phase of Experiment 1. Participants in the syllabic reduction group took longer to respond than participants in the segmental or no-reduction group. Participants, however, were not asked to respond as fast as possible. Some participants chose to do so; others waited for the sentence to finish before giving a response. The high standard deviation (SD) values reflect these different strategies. Extreme cases, that is, trials in which participants responded either too fast or too slowly, were also excluded. To do that, a linear mixed-effects model containing only participants and items as random effects and Trial Number as fixed effect was run. The residuals of this atheoretical model were computed. Based on visual inspection of a residual plot, 19 trials (0.5%) in the test phase (with residuals either below −1300 or above 3200 ms) were excluded.

**Table 2:** **RTs in ms in the test phases of Experiments 1 and 2 for clicks on targets and competitors**.

	**RT in ms**	**Segmental reduction group**	**Syllabic reduction group**	**No reduction group**
Experiment 1	Mean	1695	1769	1714
	SD	687	803	751
	Min	455	294	555
	Max	7303	8647	9107
Experiment 2	Mean	1898	1880	1730
	SD	1597	868	660
	Min	346	512	455
	Max	34467	9863	6384

#### Statistical testing

Linear mixed-effects models were used to analyze the click responses (accuracy^1^ and RT^2^) and the eye movement^3^ data on the experimental trials (the /b/-targets and the CVC-targets). To account for the categorical nature of the accuracy data, we used a logistic regression model for these data (cf. Dixon, 2008; Jaeger, 2008). The eye-tracking data were transformed into fixation proportions using the empirical logit function. Participants and Items were entered in the model as random factors including random slopes for Items. Group served as fixed effect. The segmental reduction condition (/b/-words) and the syllabic reduction condition (CVC-words) were analyzed independently. This is because a comparison between these two word sets is difficult: Both had to conform to different phonological constraints and could hence not be balanced on other variables (such as word length, lexical frequency, etc.). We therefore focus on the comparison of how the different groups recognize each word set independently (a one-factorial design with three levels: exposed to /b/-reductions, exposed to vowel-deletions, and not exposed to reductions). Trial Number was entered as another fixed effect with values centered around zero in the models for the accuracy and RT data. This variable was added to account for additional variance, as task performance often improves over the course of an experiment. The results for Trial Number, however, will not be reported below. Thus, we tested whether RTs, accuracy scores and target preference (as determined by the difference between proportion of target and competitor fixations) for the reduced words were influenced by the fixed effect of Group. That is, we examine whether the groups differ in how fast and accurately they recognize the reduced /b/-words and the vowel-deleted words and whether they show different target-competitor preferences when they process reduced words. The control group was always mapped on the intercept, so that the analysis gives two regression weights for the factor Group, one for the difference between the control group and the segmental reduction group and one for the difference between the control group and the syllabic reduction group. For the eye-tracking analyses, we had no a priori expectations about when effects would occur. We therefore analyzed the fixation data at all time points, using sliding 200 ms time windows from 200 to 1500 ms after target onset starting at every 100 ms.

#### Test phase

**Figure 2:**
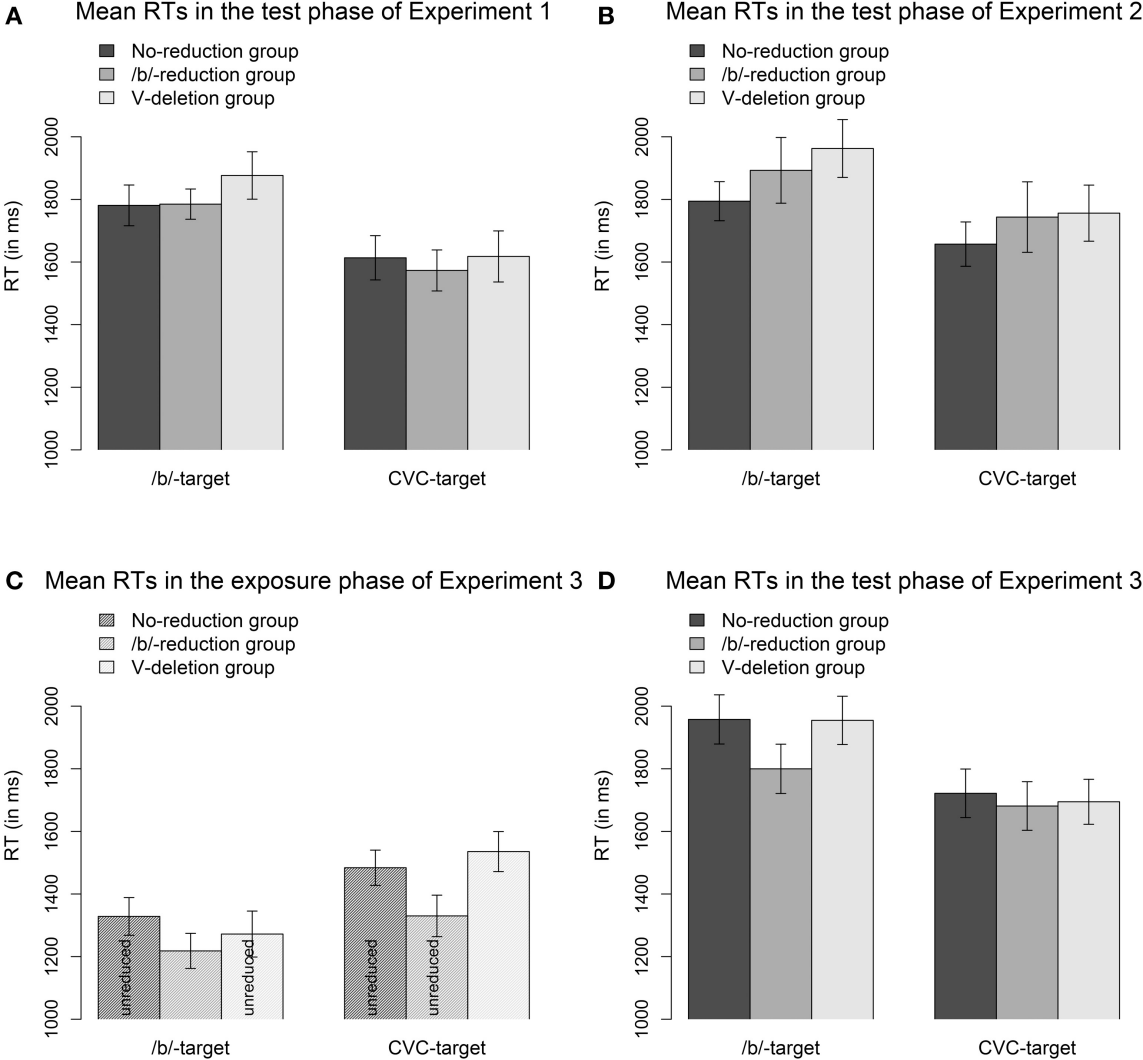
**Mean RTs and SEs in the test phases of Experiment 1 (A), Experiment 2 (B), and Experiment 3 (D) and in the exposure phase of Experiment 3 (C)**. In the test phases, the /b/-words and CVC-words were reduced for all groups. In the exposure phases, the /b/-words were reduced only for the /b/-reduction group (segmental reduction group) and the CVC-words were reduced only for the V-deletion group (syllabic reduction group).

***Reaction time data***. Figure 2A displays the mean RTs of all three groups for the reduced /b/-words (visual /b/-targets) and the vowel-deleted words (visual CVC-targets) in the test phase of Experiment 1. In the segmental reduction condition (/b/-targets), all three groups responded about equally fast and no significant differences between the groups emerged (*b*_Segmental reduction group_ = −17.9, *SE* = 87.5, *t* = −0.2, *p* = 0.84; *b*_Syllabic reduction group_ = 117.3, *SE* = 87.4, *t* = 1.3, *p* = 0.21). In the syllabic reduction condition (CVC-targets), there was also no main effect of Group (*b*_Segmental reduction group_ = −32.7, *SE* = 98.9, *t* = −0.3, *p* = 0.77; *b*_Syllabic reduction group_ = 1.7, *SE* = 97.4, *t* = 0.02, *p* = 0.98). That is, neither of the experimental groups responded faster than the control group to the reduced words. We thus did not observe any adaptation effects in the RT data.

**Figure 3:**
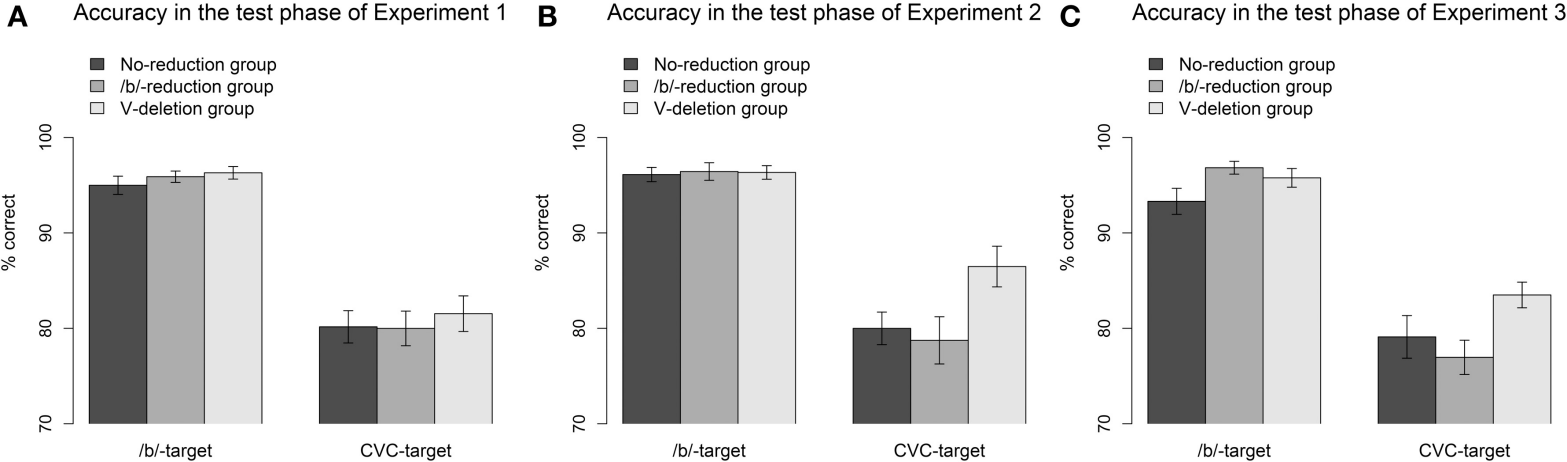
**Accuracy in % correct click responses and SEs for the reduced /b/-words (visual /b/-targets) and the vowel-deleted words (visual CVC-targets) in the test phases of Experiment 1 (A), Experiment 2 (B), and Experiment 3 (C)**.

***Accuracy data***. The accuracy data in the test phase of Experiment 1 are displayed in Figure 3A in terms of percentage of correct click responses and SEs. In the segmental reduction condition (visual /b/-targets), the main effect of Group was significant. Both the segmental reduction group (*b*_Segmental reduction group_ = 3.4, *SE* = 0.7, *p* < 0.001) and the syllabic reduction group (*b*_Syllabic reduction group_ = 2.3, *SE* = 0.5, *p* < 0.001) gave more correct responses to /b/-targets than the control group. We thus observed an adaptation effect for both experimental groups in the accuracy data for the segmental reductions.

For the syllabic reductions (visual CVC-targets), the main effect of Group was not significant (*b*_Segmental reduction group_ = 0.2, *SE* = 0.3, *p* = 0.52; *b*_Syllabic reduction group_ = 0.3, *SE* = 0.3, *p* = 0.26). That is, neither of the experimental groups differed from the control group. We thus did not observe a significant adaptation effect for either group.

**Figure 4:**
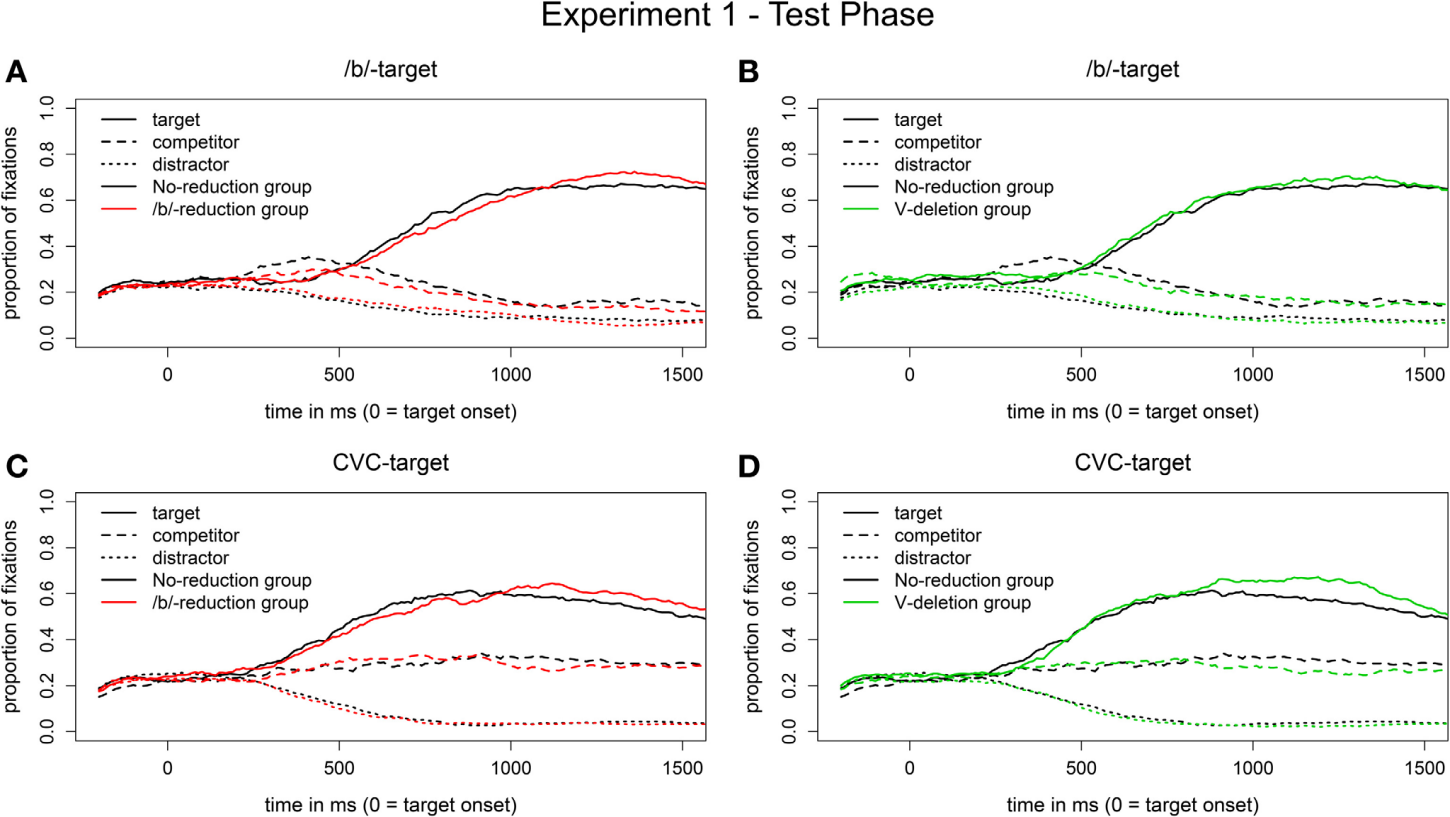
**Proportion of fixations in the segmental reduction condition [reduced /b/-words in auditory input, visual /b/-targets on screen; (A,B)] and in the syllabic reduction condition [vowel-deleted words in auditory input, visual CVC-targets on screen; (C,D)] in the test phase of Experiment 1**.

***Eye movement data***. The eye movement patterns for the segmental reduction condition (visual /b/-targets) of the two experimental groups compared to the no-reduction control group are displayed in Figures 4A,B. Early on, in a descriptive time window from 200 to 500 ms after target onset, the control group (represented by black lines) looks more often to the competitors (dashed lines) when hearing a reduced /b/-word than the segmental reduction group (in red, Figure 4A) or the syllabic reduction group (in green, Figure 4B). From around 500 ms onwards, all three groups show a similar preference for the /b/-targets (solid lines).

Statistical analyses considered time windows of 200 ms length which started at 200 ms after target onset and were then shifted by 100 ms (i.e., the following time windows were analyzed: 200–400, 300–500, 400–600, …, 1300–1500 ms). In the following and both subsequent experiments, only time windows showing significant effects are reported. If several consecutive 200 ms time windows were significant (e.g., the time windows 200–400 and 300–500 ms), the values reported are those for the accumulated time window.

The difference in target-competitor preference between the segmental reduction group and the control group did not reach significance. The main effect of Group, however, was marginally significant for the syllabic reduction group in the time window from 300 to 500 ms after target onset (*b*_Syllabic reduction group_ = 0.6, *SE* = 0.3, *t* = 1.9, *p* = 0.06). That is, we observed a weak adaptation effect for the syllabic reduction group in the segmental reduction condition, hence a weak generalization of learning across reduction types.

Figures 4C,D display the corresponding eye movement data for the syllabic reduction condition (visual CVC-targets). In the first 900 ms after target onset, all three groups show a very similar pattern for the vowel-deleted words. Only later, the two experimental groups have descriptively a greater target preference for the CVC-targets than the control group.

Statistical analyses did not reveal a significant difference between the control group and the segmental reduction group, but revealed that the main effect of Group was significant in the time window from 1100 to 1400 ms for the syllabic reduction group (*b*_Syllabic reduction group_ = 0.9, *SE* = 0.4, *t* = 2.2, *p* < 0.05). In this time window, the syllabic reduction group had a greater target-competitor preference for the CVC-words than the control group. For the syllabic reduction group, we thus found an adaptation effect.

### Discussion

In Experiment 1, we found adaptation effects for both the segmental and the syllabic reductions. Learning about segmental reductions was evident in the accuracy data but not in the eye-tracking data. For the syllabic reductions, this pattern was reversed: A learning effect was found in the eye-tracking data but not in the accuracy data. Moreover, there was also evidence of generalization of learning across reduction types. Generalization across reduction types, however, was only found in one direction: learning about vowel deletions generalized to /b/-reductions, as shown by the accuracy data and the eye movement data for the segmental reductions. In contrast, learning about /b/-reductions did not generalize. That is, the segmental reduction group could not apply their experience with reductions to the vowel-deleted words.

The learning effects found in Experiment 1 seem somewhat weak. An explanation for this may be that the potentially reduced words in the exposure phase were not highly predictable. Participants did not see the potentially reduced words on the computer screen during the exposure phase and these words appeared early in the sentences, which were in fact designed to predict the targets (e.g., in *Pas in een [b]/[m]inderij wordt een boek of **tijdschrift** afgemaakt*, the target ***tijdschrift*** is predictable and the potentially reduced word *[b]/[m]inderij* is not). Participants may therefore not have been able to predict potentially reduced words. Having information about the upcoming reduced words in advance could however facilitate learning. Jesse and McQueen (2011) found that adaptation to ambiguous fricatives did not take place if those fricatives occurred at the onset of a word presented in isolation. They concluded that lexical information likely has to be available when the ambiguous sound is initially being processed. The present study investigates adaptation to another form of deviation, which also occurs at the beginning of the words. Predictable sentence contexts may provide sufficient cues about the upcoming words so that adaptation may be possible. Experiment 2 was run to test this hypothesis.

## Experiment 2

Experiment 2 tested whether providing additional information about the reduced words in the exposure phase might strengthen the learning effects found in Experiment 1. Therefore, we changed the exposure sentences for the experimental words, leaving the filler sentences for the /m/-words and the consonant-cluster words intact. The sentence contexts now predicted the potentially reduced words. To avoid the orthographic versions of the reduced words appearing twice on the screen, the clicking task was not used in the exposure phase. Instead, participants simply listened to the exposure sentences and were asked to answer questions about the content of some of the filler sentences (those containing /m/- or CC-words).

The test phase was kept the same as in Experiment 1, apart from minor changes in three sentences (see Methods section). Further purposes of Experiment 2 were to replicate the generalization effect from vowel-deleted words to reduced /b/-words found in Experiment 1 and to test whether, with predictable sentences, a generalization effect in the other direction (from reduced /b/-words to vowel deletions) might occur.

### Methods

#### Participants

Sixty Dutch participants of the Max Planck Institute's subject pool, none of whom had participated in Experiment 1, were paid for their participation. All had normal hearing and normal or corrected-to-normal vision.

#### Design

The design was similar to that in Experiment 1. The main difference was a change in task during the exposure phase, where participants had to answer questions regarding the content of some of the reduction-free sentences without their eye movements being tracked.

#### Materials

As in Experiment 1, the exposure and the test phases consisted each of 96 trials (48 experimental trials containing either /b/-words or CVC-words and 48 filler trials containing either /m/-words or CC-words). While for the fillers the same exposure sentences as in Experiment 1 were used, new exposure sentences were generated for the experimental conditions (the potentially reduced /b/-words and the vowel-deleted words). The critical words now appeared toward the end of the sentences (e.g., *Als een manuscript gedrukt is, moet het naar de [b]/[m]inderij*. “When a manuscript is printed, it has to go to a book binder”) and were predicted by the semantic context (see cloze test below). The materials for the test phase were taken from Experiment 1. Only three target words were changed slightly (*bankier* “banker” → *bankiers* “bankers,” *benauwen* “to oppress” → *benauwd* “sultry,” *coulisse* “wing [of theater stage]” → *coulissen* “wings, pl.”) so that it was possible to create more natural sentences for the exposure phase.

#### Cloze tests

Cloze tests were run to check the degree of predictability of the potentially reduced words in the exposure sentences. The 48 sentences were presented in a randomized order with the critical word replaced by a gap. Participants were instructed to complete these sentences with one word. They were asked to type in at least one answer but had the possibility to give up to seven. After typing in their answer(s), participants saw the same sentence again completed with the corresponding /b/- or CVC-target. They were asked to rate how well the proposed solution completed the sentence context on a scale from 1 (“Word does not fit at all”) to 7 (“Word fits perfectly”). The cloze tests were self-paced; it took participants 15 to 30 min.

An initial test with eighteen Dutch native speakers of the Max Planck Institute's subject pool, who had not participated in Experiment 1, showed that for some sentences the target word was mentioned in less than 25% of cases. These were improved if possible. A second version of the cloze test was run with 19 new Dutch participants. We analyzed the percentages of mentioned target words in the sentence completion task and the mean ratings for the targets in the rating task. The critical /b/-words were mentioned in 36% of the cases, while the critical CVC-words were mentioned in 51% of the cases. This difference does not reflect a frequency effect, as the /b/-targets are more frequent than the CVC-targets (see Tables S1, S2). But it can possibly be explained by the higher constraints on the initial selection of the /b/-words. Only /b/-words were chosen which had a nasal in third position and for which a /m/-initial competitor with as much onset overlap as possible existed. Similar constraints on the CVC-words were less strong, as the consonants in first and third position could vary. Although participants did not come up with our solutions in many cases, they rated those solutions very highly on average: On a scale from 1 to 7, with higher ratings meaning better fits, participants rated the /b/-targets 6.1 and the CVC-targets 6.3 on average.

#### Stimulus construction

The new exposure sentences were recorded by the same female Dutch speaker who provided the stimuli for Experiment 1. The reduced stimuli were created in the same way as described in Experiment 1.

#### Procedure

Participants were tested in a sound-proof booth. They were told that the experiment consisted of two parts. For the first part, they were asked to listen to sentences that were presented over headphones and to answer questions regarding the content of these sentences (by clicking on one out of two suggested solutions) that might appear at random points in time on the screen.

Each exposure sentence was preceded by 500 ms of silence and followed by 2000 ms of silence. If a question and two possible solutions were to appear on the screen (after six /m/-word sentences and after six CC-word sentences, i.e., in ^1^/_8_th of the exposure trials), they followed the auditory stimulus immediately. After participants had clicked on the screen, it took 1000 ms before the next exposure trial started. The order in which the exposure sentences were played was randomized for each participant individually. Participants had the opportunity to take a break approximately halfway through the experiment, after the 50th stimulus (out of 96).

The procedure of the test phase was identical to the one in Experiment 1, except that eye movements were monitored using an SR Research EyeLink II, sampling at 500 Hz. An experimental session took approximately 30 min.

### Results

#### Exclusion criteria

The same criteria as in Experiment 1 were applied for trial exclusion. This led to the exclusion of 2.2% of the data due to fixations outside of the screen area and of another 1.3% due to failure to click on the target or the potentially confusable competitor. An additional 0.5% of trials were discarded because they were considered to be RT outliers (with residual values either below −2300 or above 3100 ms). For the eye-tracking data, we analyzed the data from the better eye of the participants (i.e., the eye that showed less error in the validation of the calibration of the eye-tracker).

#### Exposure phase

Participants of all groups hardly made errors in the comprehension questions of the exposure phase. Each group obtained a score of 99% correct responses.

#### Test phase

***Reaction time data***. The lower part of Table 2 shows the descriptive statistics for the RT data in the test phase of Experiment 2. The mean RTs and their SEs of all three groups for the reduced /b/-words (visual /b/-targets) and vowel-deleted words (visual CVC-targets) are displayed in Figure 2B. The no-reduction control group seems to respond slightly faster than the two experimental groups in both the segmental reduction condition (/b/-targets) and the syllabic reduction condition (CVC-targets). However, the main effect of Group was not significant in either condition (/b/-targets: *b*_Segmental reduction group_ = 118.2, *SE* = 127.0, *t* = 0.9, *p* = 0.38; *b*_Syllabic reduction group_ = 191.2, *SE* = 130.2, *t* = 1.5, *p* = 0.15; CVC-targets: *b*_Segmental reduction group_ = 93.0, *SE* = 121.5, *t* = 0.8, *p* = 0.43; *b*_Syllabic reduction group_ = 123.2, *SE* = 121.5, *t* = 1.0, *p* = 0.33). As there was no main effect of Group in the RT data indicating that one or both of the experimental groups responded faster to the reduced targets than the control group, we did not observe any adaptation effect.

***Accuracy data***. Figure 3B shows the accuracy data in percentages correct responses and SEs of all three groups for the reduced /b/-words (visual /b/-targets) and vowel-deleted words (visual CVC-targets). All three groups performed near ceiling in the segmental reduction condition (/b/-targets). There was no difference between the groups (*b*_Segmental reduction group_ = −0.4, *SE* = 0.5, *p* = 0.44; *b*_Syllabic reduction group_ = −0.6, *SE* = 0.5, *p* = 0.22) indicating that the experimental groups did not respond more accurately than the control group. We thus did not observe an adaptation effect in the accuracy data for the segmental reduction condition.

In the syllabic reduction condition (CVC-targets), the main effect of Group was significant for the syllabic reduction group (*b*_Syllabic reduction group_ = 0.9, *SE* = 0.3, *p* < 0.01) but not for the segmental reduction group (*b*_Segmental reduction group_ = 0.2, *SE* = 0.3, *p* = 0.54). That is, only the syllabic reduction group gave more correct answers when hearing a vowel-deleted word than the no-reduction control group. We thus observed a learning effect for the syllabic reduction group, but no generalized learning effect for the segmental reduction group.

**Figure 5:**
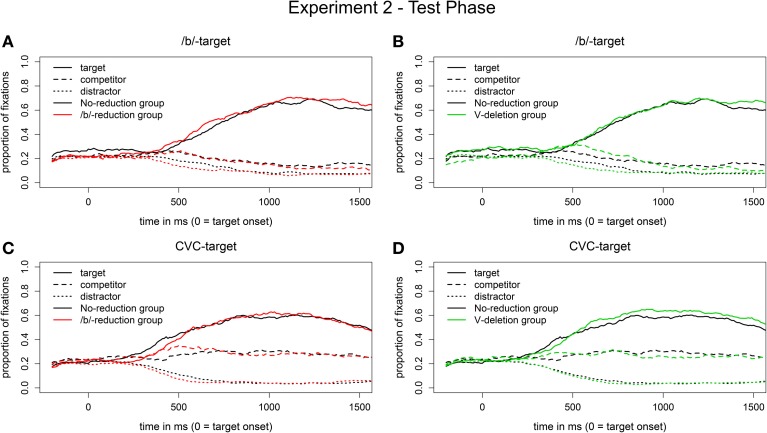
**Proportion of fixations in the segmental reduction condition [reduced /b/-words in auditory input, visual /b/-targets on screen; (A,B)] and in the syllabic reduction condition [vowel-deleted words in auditory input, visual CVC-targets on screen; (C,D)] in the test phase of Experiment 2**.

***Eye movement data***. Figures 5A,B shows the eye-movement patterns in the segmental reduction condition (visual /b/-targets) for the segmental reduction group (in red) and the syllabic reduction group (in green) compared to the no-reduction control group (in black). All three groups behave very similarly when hearing reduced /b/-words. There was indeed no main effect of Group. That is, we did not observe a learning effect for the segmental reduction condition in the eye-tracking data.

The corresponding eye movement data for the syllabic reduction condition (visual CVC-targets) are displayed in Figures 5C,D. Statistical analysis revealed a marginal main effect of Group (*b*_Segmental reduction group_ = −0.6, *SE* = 0.3, *t* = −2.0, *p* = 0.06) in the time window from 200 to 500 ms after target onset. The segmental reduction group had a smaller preference for the CVC-targets over the CC-competitors than the control group in this time window. We thus observed a marginal inhibitory effect for the segmental reduction group, given that participants in this group, who had experience with another type of reduction, showed a smaller target preference than participants in the control group, who had not been exposed to any reductions. Furthermore, no learning effect was found for the syllabic reduction group.

### Discussion

Experiment 2 was conducted to replicate the findings of Experiment 1 and to test whether predictability of the reduced words during exposure enhances the learning effects. As in Experiment 1, adaptation was observed in the syllabic reduction condition. Contrary to the previous experiment, it was found in the accuracy data, not in the eye-tracking data. The pattern of target and competitor fixations for the syllabic reduction group, however, was in the expected direction (see Figure 5D). We did not replicate the learning effect for segmental reductions found in the accuracy data in Experiment 1. Neither could we replicate the generalized learning effect for the syllabic reduction group for vowel-deletions to /b/-reductions (that was also evident in the accuracy data of Experiment 1). Another generalization effect emerged, however. In contrast to Experiment 1, the segmental reduction group differed from the control group when dealing with vowel deletions. In the eye-tracking data, they showed a smaller target-competitor preference for CVC-targets. That is, even though they did not show a learning effect for /b/-reductions, participants in the segmental reduction group seemed to be hindered by their exposure to /b/-reductions and struggled more with recognizing the vowel-deleted words than the control group.

In Experiments 1 and 2, we found learning effects for repeated /b/-reductions and vowel-deletions. At this point, we cannot say whether these effects are truly word-specific, meaning that they arose because the reduced forms were stored after their first encounter in the mental lexicon and then accessed again as they were encountered the second time in the test phase. The observed effects could also have arisen because of rule abstraction. To determine which mechanism is responsible for the learning effects found for repeated reduced words in Experiments 1 and 2, we tested whether learning can generalize to other words of the same reduction type in Experiment 3. If there is no or only weak evidence for generalized learning, then the effects found for repeated words are very likely to be word-specific. In contrast, if there is strong evidence for generalized learning, then the effects found for repeated words are likely due to abstraction processes.

The null result for the segmental reductions in Experiment 2 suggests that predictable sentences alone might not be enough to induce a stable adaptation effect. In Experiment 3, we therefore combined aspects of the exposure phase of Experiment 1 (eye-tracking with printed words on the screen) with aspects from Experiment 2 (predictable sentence context). This procedure should render the reduced target words highly predictable, which in turn could lead to a strong learning effect. Using eye-tracking in the exposure phase can tell us whether participants actually make use of the sentence context (i.e., they might already look at the target word before it is mentioned).

## Experiment 3

In Experiment 3, we tested whether learning about reductions can generalize across words (within a reduction type). To that end, new /b/-words and new CVC-words were selected for the exposure phase and new exposure sentences were created in which those words were predictable. In the exposure phase, participants had to click on the potentially reduced /b/-target and CVC-target words, while their eye-movements were recorded. The test phase was the same as in Experiment 1. Importantly, the target words used in the test phase did not occur in the exposure phase. Apart from the generalization of learning within a reduction type, Experiment 3 again tests generalization of learning across reduction types and aims to replicate and extend the results from Experiment 1 on this issue.

### Methods

#### Participants

Sixty Dutch participants of the Max Planck Institute's subject pool, none of whom had participated in the previous experiments, took part for a small remuneration. All reported normal hearing and normal or corrected-to-normal vision.

#### Design

The design was very similar to the one of Experiment 1, except for changes in the exposure phase. Predictable exposure sentences were created for new potentially reduced /b/-words and vowel-deleted words which served as target words in an eye-tracking paradigm. That is, participants had to click on the orthographic form of these words while their eye movements were recorded. The test phase was the same as in Experiment 1. Due to the changes in the exposure phase, the targets in the test phase were new to participants and not repeated as in Experiments 1 and 2 (see Table 3).

**Table 3:** **Experimental design and types of stimuli in Experiment 3**.

	**Trial type**	**Canonical word-form**	**Segmental reduction group**	**Syllabic reduction group**	**Control group**
			**/b/ → [m]**	**Full vowel deletion**	**No reduction**
Exposure phase	Experimental	/bɑndit/	[**m**ɑndit]	[bɑndit]	[bɑndit]
	Filler	/mɑtros/	[mɑtros]	[mɑtros]	[mɑtros]
	Experimental	/kanal/	[kanal]	[**kn**al]	[kanal]
	Filler	/knɔflok/	[knɔflok]	[knɔflok]	[knɔflok]
Test phase	Experimental	/bɪndərεɪ/		[**m**ɪndərεɪ]	
	Filler	/murɑs/		[murɑs]	
	Experimental	/parat/		[**pr**at]	
	Filler	/xlɑns/		[xlɑns]	

*Reduced segments are marked bold. The potentially reduced /b/-initial words and the vowel-deleted words of the exposure phase were not repeated in the test phase*.

#### Materials

As in Experiments 1 and 2, the exposure and the test phases consisted each of 96 trials (48 experimental trials containing either /b/-words or CVC-words and 48 filler trials containing either /m/-words or CC-words). The exposure sentences for the /m/-words and the CC-words were the same as in Experiment 1. The exposure sentences for the potentially reduced /b/-words and vowel-deleted words were constructed anew. These critical words appeared again toward the end of the sentences and were predicted by the semantic context.

For the selection of the 24 exposure /b/-targets and the 24 exposure CVC-targets, the same constraints applied as for the respective targets of the test phase. The criteria for the selection of their “competitors” were less strict. These only overlapped in the initial consonantal part for reduced forms, but were additionally matched on word class (e.g., *bandiet* “bandit” would be reduced to [mɑndit] and would compete for recognition with [mirakəl] *mirakel* “miracle”; *kanaal* “canal” would be reduced to [knal] and would compete with [knεxt] *knecht* “servant”). The materials for the test phase were taken from Experiment 1.

#### Stimulus construction

The new exposure sentences were recorded by the same female Dutch speaker as in Experiments 1 and 2. The reduced stimuli were created as described in Experiment 1.

#### Procedure

The procedure was similar to Experiment 1 except for changes in the exposure phase, in which participants had to click on the potentially reduced word that was predictable from the sentence context. The computer display always showed a /b/-word, a /m/-word, a CVC-word and a consonant-cluster word on the screen. Exposure and test displays differed only in the phonological similarity of target and competitor words which were more similar in the test phase (e.g., exposure trial: *bandiet* vs. *mirakel*; test trial: *binderij* vs. *minderjarig*). An experimental session took approximately 25 min.

### Results

#### Exclusion criteria

Trials were excluded based on the same criteria as used in Experiments 1 and 2. Due to fixations outside of the screen, 2.6% of the trials were removed. Another 0.6% were discarded due to failure to click on the target or the potentially confusable competitor. Fifteen trials (0.3%) in the exposure phase (with residuals either below −1100 or above 2500 ms) and 29 trials (0.5%) in the test phase (with residuals either below −1700 or above 2800 ms) were considered to be RT outliers and hence excluded. For the eye-tracking results, the data of the participants' right eye were analyzed.

An overview of the accuracy data in the exposure and test phases can be found in Table 4. In the exposure phase, practically no errors were made. In the test phase, we again observe a high percentage of errors for the vowel-deleted words in all three groups. Table 5 displays the descriptive statistics for the RT data in the exposure and test phases. All three groups took longer to give a click response in the test phase (where the sentence context was neutral and the words on the screen were quite similar to each other) than in the exposure phase (where they could use the sentence context to predict the target word). The negative minima for RTs in the exposure phase confirm that the target words in this phase were indeed predictable, as some participants responded even before target onset.

**Table 4:** **Accuracy data of the exposure and test phases of Experiment 3**.

**% Click responses**		**Segmental reduction group**			**Syllabic reduction group**			**No reduction group**		
		**Target**	**Comp**.	**Distr**.	**Target**	**Comp**.	**Distr**.	**Target**	**Comp**.	**Distr**.
Exposure	/b/-word	99.4	0.0	0.0	99.4	0.0	0.0	99.6	0.0	0.0
	CVC-word	99.6	0.0	0.0	99.4	0.0	0.4	99.8	0.0	0.0
Test	/b/-word	95.8	3.1	0.0	95.2	4.2	0.0	92.3	6.7	0.2
	CVC-word	75.8	22.7	0.0	83.3	16.5	0.0	79.0	20.8	0.0

**Table 5:** **RT in ms in the exposure and test phases of Experiment 3 for clicks on targets and competitors**.

**RT in ms**	**Segmental reduction group**		**Syllabic reduction group**		**No reduction group**	
	**Exposure**	**Test**	**Exposure**	**Test**	**Exposure**	**Test**
Mean	1280	1754	1429	1876	1418	1889
SD	610	736	761	947	644	955
Min	−40	554	−40	430	−39	677
Max	7374	8015	7060	10854	6419	9599

#### Exposure phase

**Figure 6:**
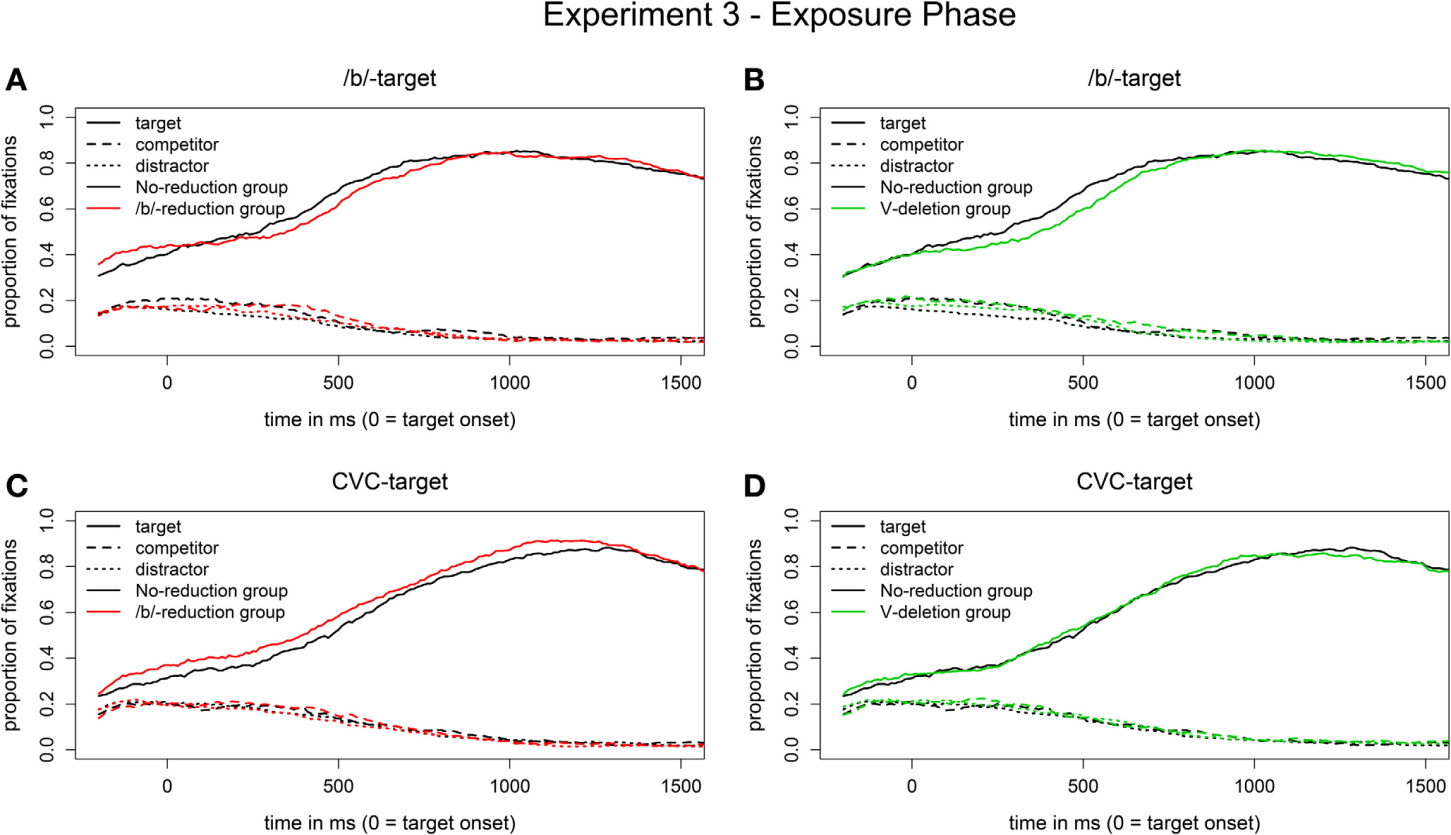
**Proportion of fixations in the segmental reduction condition [visual /b/-targets; (A,B)] and in the syllabic reduction condition [visual CVC-targets; (C,D)] in the exposure phase of Experiment 3**. The /b/-words were reduced only for the /b/-reduction group (segmental reduction group) and the CVC-words were reduced only for the V-deletion group (syllabic reduction group).

The /b/-words were reduced only for the segmental reduction group and the CVC-words were reduced only for the syllabic reduction group. There were virtually no errors (see Table 4). Moreover, in contrast to previous results (Poellmann et al., under revision), there was no consistent effect of reduction, neither in RTs nor in the eye-tracking data (see Figures 2C, 6; the main effect of Group was not significant for the groups who heard reduced forms indicating that they did not have more difficulties in recognizing the targets than the control group). The data from the exposure phase reflect that the target words were predictable, as participants in all groups already showed a preference for the target before it was mentioned (see the time windows from −200 to 0 ms in Figure 6). Apparently, the words in the exposure phase were recognized efficiently whether they were reduced or not.

#### Test phase

***Reaction time data***. Figure 2D displays the mean RTs and SEs of all three groups for the reduced /b/-words (visual /b/-words) and the vowel-deleted words (visual CVC-words) in the test phase of Experiment 3. The segmental reduction group seems to respond somewhat faster to the reduced /b/-words, while all three groups seem to respond about equally fast to the vowel-deleted words.

Statistical analyses did not show a main effect of Group—neither in the segmental reduction condition (/b/-targets: *b*_Segmental reduction group_ = −177.1, *SE* = 108.5, *t* = −1.6, *p* = 0.12; *b*_Syllabic reduction group_ = −15.2, *SE* = 106.4, *t* = −0.1, *p* = 0.92) nor in the syllabic reduction condition (CVC-targets: *b*_Segmental reduction group_ = −7.8, *SE* = 102.8, *t* = −0.1, *p* = 0.92; *b*_Syllabic reduction group_ = 11.7, *SE* = 103.0, *t* = 0.1, *p* = 0.92)—indicating that all groups responded equally fast to both types of target words. That is, the groups experienced with reduced forms did not respond faster than the less experienced control group. We thus did not observe any adaptation in the RT data.

***Accuracy data***. The accuracy data in terms of percentage correct responses and their SEs of all groups can be found in Figure 3C. Both experimental groups seem to give more accurate responses to reduced /b/-words (visual /b/-words) than the control group. For the vowel-deleted words (visual CVC-words), only the syllabic reduction group seems to respond more accurately than the control group. This, however, was not confirmed by statistical analyses. The main effect of Group was not significant either in the segmental reduction condition (/b/-targets: *b*_Segmental reduction group_ = 0.8, *SE* = 0.5, *p* = 0.16; *b*_Syllabic reduction group_ = 0.1, *SE* = 0.5, *p* = 0.90) or in the syllabic reduction condition (CVC-targets: *b*_Segmental reduction group_ = −0.4, *SE* = 0.3, *p* = 0.16; *b*_Syllabic reduction group_ = 0.4, *SE* = 0.3, *p* = 0.21). That is, neither of the experimental groups gave more correct answers to the reduced targets than the control group. We thus did not observe any adaptation effects in the accuracy data.

**Figure 7:**
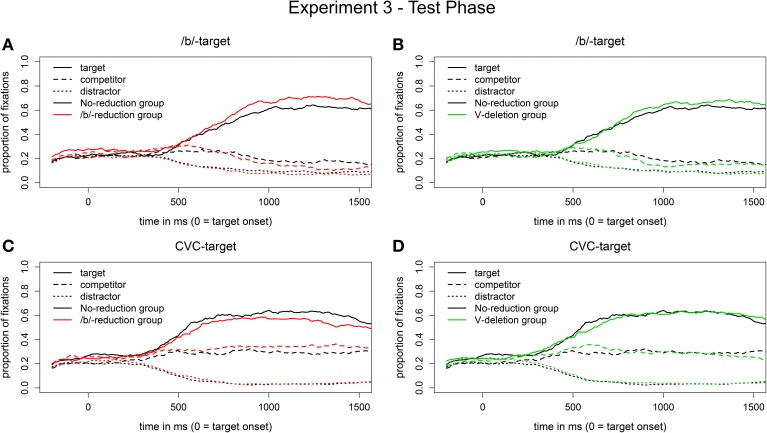
**Proportion of fixations in the segmental reduction condition [reduced /b/-words in auditory input, visual /b/-targets on screen; (A,B)] and in the syllabic reduction condition [vowel-deleted words in auditory input, visual CVC-targets on screen; (C,D)] in the test phase of Experiment 3**.

***Eye movement data***. Figures 7A,B display the eye movement pattern of the two experimental groups plotted against the patterns of the control group (in black) for the segmental reduction condition. Both experimental groups show a greater preference for the target over the competitor for the reduced /b/-words than the control group, descriptively from around 700 ms onwards (when the colored lines diverge from the black lines). This difference is bigger for the segmental reduction group (in red).

The main effect of Group reached significance only for the segmental reduction group (*b*_Segmental reduction group_ = 1.0, *SE* = 0.4, *t* = 2.4, *p* < 0.05) from 1100 ms onwards. That is, the segmental reduction group, but not the syllabic reduction group, outperformed the control group on the reduced /b/-words. We thus observed a within-reduction-type generalization effect in the segmental reduction condition.

The corresponding eye-movement data for the syllabic reduction condition are displayed in Figures 7C,D. The segmental reduction group (in red) shows a smaller target-competitor preference for the CVC-targets than the control group, descriptively from 500 ms onwards. The syllabic reduction group shows a similar pattern from 500 to 700 ms after target onset.

Statistical analyses showed a marginally significant main effect of Group only for the segmental reduction group (*b*_Segmental reduction group_ = −0.9, *SE* = 0.5, *t* = −2.0, *p* = 0.06) in the time window from 1200 to 1500 ms. That is, the segmental reduction group but not the syllabic reduction group had a significantly smaller target preference for the vowel-deleted words than the control group. We thus did not observe a learning effect for the syllabic reduction group and found a marginal inhibitory effect for the segmental reduction group. Participants in the latter group seem to be hindered by their prior exposure to another reduction type.

### Discussion

The aim of Experiment 3 was to test whether learning about reductions can generalize within and across reduction types. In the exposure phase, listeners were provided with predictive sentence contexts and with orthographic information about the critical words, as they saw the orthographic forms of the potentially reduced words on the computer screen. The results from the exposure phase did not show any effects of reduction. That is, neither the segmental reduction group nor the syllabic reduction group were slowed down or had a smaller target preference when hearing reduced forms. This is very likely due to the predictive sentence context. Participants were already expecting the target and looking at it before it was actually mentioned. Hearing it then in reduced form did not disturb the recognition process any more. Note that these data apparently are in contrast with the data of Brouwer et al. (2013), who found that even predictable words suffer from reduction costs. The difference, however, might be due to the stimulus material, with our material being constructed to allow prediction of the target word, while Brouwer et al. used materials from a speech corpus. For reduced words which were particularly predictable, they also observed less reduction costs.

In the test phase, we found clear evidence for generalization of learning within reduction type for the segmental reduction group in the eye-tracking data. No such generalization effect was found for the syllabic reduction group. Contrary to the word-specific learning effects found in Experiments 1 and 2, the within-reduction-type generalizations were stronger for /b/-reductions than for vowel-deletions.

As for generalization of learning across reduction types, we did not replicate the transfer of learning from vowel-deletions to /b/-reductions for the syllabic reduction group found in Experiment 1. There was a trend going in this direction though (see Figure 7B). However, we replicated the marginal inhibitory effect of the segmental reduction group found in Experiment 2. That is, the segmental reduction group did not benefit from its exposure to /b/-reductions and had instead slightly greater problems in recognizing vowel-deletions than the no-reduction control group.

## General discussion

The present study investigated whether and how listeners can adapt when they encounter reduced word forms. In the introduction, we argued for a continuum of possible adaptation mechanisms that are more or less general. At the specific end, listeners may only adapt to exactly the same words. A more general adaptation would allow generalization to other words of the same or a similar reduction type. Experiments 1 and 2 tested learning effects for repeated segmental and non-morphemic syllabic reductions. Experiment 3 examined whether these learning effects were word-specific by testing whether learning about these reductions generalizes to new words of the same reduction type (within-reduction-type generalization). All three experiments investigated whether experience with one reduction type helps the listener in dealing with another reduction type (across-reduction-type generalization).

Experiments 1 and 2 showed evidence of learning for repeated vowel-deletions but, surprisingly, far less so for repeated /b/-reductions. In contrast, Experiment 3 revealed a strong within-reduction-type generalization effect in the eye-tracking data for the /b/-reductions that was not found for the vowel-deletions. In Experiments 2 and 3, the segmental reduction group further showed a marginal inhibitory effect; they had greater difficulties than the control group dealing with unfamiliar vowel-deletions. Another pattern that was consistently observed (even though not always statistically significant) was that the syllabic reduction group made fewer errors for both the same and other vowel-deleted words (see Figures 3A,B,C, focusing on the CVC-targets, e.g., *paraat* produced as *p'raat*). Next to this reduction-specific adaptation, this group also showed generalization of learning across reduction types (from vowel-deletions to /b/-reductions). This generalization effect, however, could not always be found: It was absent in Experiment 2 where task demands in the exposure phase were low and the predictability of the reduced word was high. It was present in Experiment 1, where task demands in the exposure phase were high, but the predictability of the reduced word was low. Finally, a trend was observed again in Experiment 3, where both task demands and the predictability of the reduced word in the exposure phase were high.

The results of Experiment 3 shed further light on the learning effects found in Experiments 1 and 2. For the segmental reductions, strong generalization of learning to new reduced /b/-words was observed. This suggests that, for the /b/-reductions investigated here, recognition predominantly occurs via abstraction rules. It is therefore likely that abstraction processes also play a role in the recognition of repeated reduced /b/-words. The learning effect found for repeated reduced /b/-words in Experiment 1 thus is very likely not a word-specific adaptation. For the vowel-deletions, no generalization of learning to other vowel-deleted words was observed in Experiment 3. The adaptation effects for repeated vowel-deleted words found in Experiments 1 and 2 are therefore very likely due to storage of these reduced forms and hence are word-specific. Similarly, Hanique et al. (2013) claim that, if the absence of schwa in the prefix of Dutch past participles is due to categorical processes, these schwa-deleted forms are stored in the mental lexicon.

Lexical storage is not only useful if a listener encounters a reduced word for the first time, but may also help to build up abstraction rules for later generalization of learning to other words that show the same reduction pattern. It is therefore surprising that we did not find any benefit for repeated reduced /b/-words in Experiment 2, while we did find a benefit for repeated vowel-deleted words under the same circumstances. Furthermore, although small, such a benefit was found for repeated reduced /b/-words in Experiment 1, where participants were involved in a more active task, but where the reduced /b/-words were hardly predictable. One possible explanation for these findings is based on the difference in saliency between the two reduction types. In the vowel-deletions, an entire segment is completely deleted, whereas in the /b/-reductions the segment is only weakened. The vowel-deletions are thus more striking than the /b/-reductions and potentially are therefore less susceptible to experimental manipulations. Apparently, manipulating the preceding context to make the reduced /b/-words more predictable was not enough to draw participants' attention to that reduction type, while giving listeners a more active task might have achieved this. Learning about reductions might thus only occur if the reduction type is (made) salient enough. Note that in Experiment 3, where learning for /b/-reductions was found, listeners saw the orthographic form of the reduced /b/-words on the screen already in the exposure phase. This may have boosted the learning effect.

The within-reduction-type generalization effect found for new reduced /b/-words in Experiment 3 supports the assumption of an abstractionist mode of lexical access. For the vowel-deletions, only a hint of this generalization effect was observed (in the accuracy data). An important difference between /b/-reductions and vowel-deletions that could explain this discrepancy is input consistency. In the /b/-initial words that were to be reduced, the /b/ was always followed by a vowel and a nasal. The structure of the CVC-words was less consistent: The first consonant could be /k, x, p/, the vowel to be deleted was variable and the second consonant was either a liquid or /n/. The phonological context surrounding the reduced segment and the reduced segment itself varied thus more in the vowel-deletions than in the /b/-reductions. This input variability for vowel-deletions may have been too high for the successful generation of an abstract mapping rule. This very likely restricts generalized learning about syllabic reductions to morphemes that show a high frequency of occurrence across words.

There hence seems to be evidence for two types of adaptation: word-specific adaptation to inconsistent phonological patterns and word non-specific adaptation to consistent patterns. More general learning effects, if observed at all, were marginal. This already suggests that it is hard to apply the knowledge of one reduction type to another in case the two reduction types differ substantially. Nevertheless, we observed such a non-specific adjustment to reductions for the syllabic reduction group. Listeners in this group showed a greater tolerance to /b/-reductions than the control group. Possible factors that likely play a role in this uni-directional facilitative effect are input variability and degree of reduction. These two factors, however, are (necessarily) confounded in the present study. The vowel-deletions are both more variable in their segmental structure and more severely reduced than the /b/-reductions. Similar conditions were present in the study by Brouwer et al. (2012). Brouwer et al. focused on processing at the lexical level and selected reductions which had more onset overlap with another existing word than with their respective canonical form (e.g., the reduced form [pjutər] from *computer* is at the onset more similar to the word *pupil* than to *computer*). As a consequence, their set of reductions contained a large variety of reductions, making it unlikely that listeners could adapt to a specific form of reduction. With this set of varying reductions, they found similar facilitative effects as observed here for the group exposed to variable vowel deletions. They reported that listeners penalized acoustic mismatches between input and canonical form less strongly when listening to (strongly and therefore not regularly) reduced speech.

Instead of also observing facilitation for the segmental reduction group in dealing with vowel-deletions, we found marginal inhibitory effects. After having been exposed to consistently reduced /b/-words, the segmental reduction group did worse on the more strongly reduced vowel-deleted words than the control group. It might thus be that learning about reduction can only generalize to other reduction types that are of the same or a lesser degree of reduction but not to reduction types that show a higher degree of reduction. Another possibility is that the vowel-deletions differed in too many ways from the /b/-reductions so that it was not possible to adjust the abstract mapping rule for /b/-reductions to accommodate the variable vowel-deletions.

But why did the segmental reduction group actually differ from the control group in dealing with vowel-deletions? It might be the case that participants in the segmental reduction group expected the speaker to produce reductions only in a consistent way and to a specific degree (e.g., weakening of a segment). This might have biased them against other types of variability and the greater deviation from the canonical form that they encountered in the test phase. The control group, in contrast, had not heard any reductions in the exposure phase. In the subsequent test phase, participants in that group suddenly had to deal with many and various reduced forms. As they could not have built up abstract mapping rules, they probably resorted to flexible, non-specific adjustments, like those observed by Brouwer et al. (2012). Finally, the syllabic reduction group was already used to dealing with variable reduced forms. Participants in this group could therefore handle a consistent and less severe reduction type. How well listeners can handle new reduced forms of a different reduction type might thus also depend on listeners' expectations about a speaker's reduction style and, based on that, on the adaptation mechanisms already in use (specific abstraction rules vs. fast perceptual but non-specific adjustments).

What does this series of eye-tracking experiments tell us about possible constraints and the time-course of learning about reductions? Apparently, the reduced forms have to be noticeable, as learning effects were found for less salient reduction types only if the reduced words appeared in orthographic form on the screen (Experiment 3) or if the listener was actively involved in the task (Experiment 1), whereas this was not necessary for salient reduction types. Interestingly, the generalization effects across reduction types varied in strength across experiments, which suggests that at least some part of learning is susceptible to our experimental manipulations. Attention as measured by task involvement (Experiment 1) seems to be of greater importance than predictability (Experiment 2) in dealing with reductions. However, the combination of these two factors (Experiment 3) yielded only a trend in the expected direction.

Moreover, the time-course results suggest that the point in time when learning about reductions takes effect may depend on the specificity of the learning process. Facilitative and inhibitory generalization effects across reduction types, which are likely not specific to any segments or words in our study, were observed early in the fixation data throughout the study (from 200 to 300 ms after target onset respectively). The inhibitory effect in Experiment 3 also emerged early (around 500 ms after target onset) but reached marginal significance only late (at 1200 ms). In contrast, the effect for generalization within reduction type in Experiment 3 was quite late (starting at 1100 ms after target onset). The word-specific effect found in Experiment 1 was equally late. The former may be explained with the kind of mapping procedure participants have to apply. Listeners learned that this particular speaker was likely to pronounce a /b/ as an [m] and hence that an existing sound ([m]) mapped onto two categories for that speaker (/m/ and /b/). Their perception of an [m] might therefore have shifted from judging it as /m/ in most cases to judging it as /m/ in 80% and as /b/ in 20% of the cases. With this kind of learning, an initial signal-driven hypothesis strongly favors the canonical form, and only when later-arriving segments rule that form out can the learning take effect. Therefore, as soon as listeners receive evidence that a particular sound can map onto more than one category, the rule-based learning process likely needs more time to take effect. Similar reasoning can be applied to word-specific learning. At some point in time, the activation of *Parijs* “Paris” has to win over the activation of *prijs* “price” when hearing the reduced speech input *P'rijs*. Initially, the activation of *prijs* is likely to be stronger as this meaning is encountered much more frequently. Speaker-specific information (e.g., on the tendency of this speaker to reduced words like *Parijs*) then has to kick in and shift the weights in favor of the candidate *Parijs*. This may not happen immediately.

As stated before, all measures in these experiments (RT, accuracy, eye movements) could reflect improvements in spoken-word recognition due to adaptation to deviant pronunciations. However, as we did not push participants to click as fast as possible, it is perhaps not surprising that RTs did not show adaptation effects in any of the experiments. The eye-tracking data may be the more sensitive measure of adaptation because the fixation behavior does not necessarily entail conscious decision processes (unlike the click responses). Note that although we found a word-specific learning effect for vowel-deleted words in Experiment 2 in the accuracy data but not in the eye-tracking data, the eye-tracking data did show a non-significant trend in the expected direction. Note also that there were fewer participants in Experiment 2 than in Experiment 1 (60 vs. 75). It is thus possible that with more participants both measures might have shown significant effects.

Finally, it has to be noted that the learning effects (i.e., the differences between groups) were rather subtle. As stated at the beginning of the introduction, we investigated *whether* and *how* adaptation plays a role in the recognition of reduced forms. As discussed, the small learning effects speak for both episodic storage and abstraction in response to different challenges posed by different forms of reduction (answering the *how* question). Additionally, the group differences were consistently small, despite a reasonably large N (at least 60 participants in each experiment). Adaptation effects of considerable magnitude have been found with much smaller groups in conceptually similar experiments (e.g., Reinisch et al., 2013). This seems to indicate that short-term adaptation is only one piece of the puzzle concerning how we are able to understand speech despite considerable phonological reduction (answering the *whether* question).

## Conclusion

The present study provided evidence that listeners use a wide variety of adaptation mechanisms when dealing with reduced forms. Word-specific learning effects showed that reduced forms are sometimes stored as such in the mental lexicon. If possible, that is, if the input was sufficiently consistent, abstraction rules were generated based on the reduced speech input and applied to new reduced words. In the setting of the present study, this was only successful for new words of the same reduction type. If the input was too inconsistent, listeners showed perceptual flexibility and were able to deal with various reduction types. The interplay of abstraction processes and perceptual adjustments may come at a cost if abstract mapping rules are already in place. The perceptual system might then not be flexible enough to allow rapid accommodation to inconsistent reductions. To conclude, both episodic and abstractionist modes of lexical access, as well as perceptual flexibility, play a role in recognizing reduced word forms.

## Author contributions

This work is part of Katja Poellmann's Ph. D. project.

### Conflict of interest statement

The authors declare that the research was conducted in the absence of any commercial or financial relationships that could be construed as a potential conflict of interest.

## Footnotes

^1^lmer(Accuracy ~ Group + scale(Trial_Number, scale = F) + (1 |Participant) + (1 + Group |Item), data = test, subset = Trial_Type == “test_b”, family = binomial).

^2^lmer(RT ~ Group + scale(Trial_Number, scale = F) + (1 |Participant) + (1 + Group |Item), data = test, subset = Trial_Type == “test_b”).

^3^lmer((empLogit(targetProp, 20) – empLogit(compProp,20)) ~ Group + (1 |Participant) + (1 + Group |Item), data = test, subset = Trial_Type = = “test_b”).
