# Supplementary material for: Use what you can: storage, abstraction processes, and perceptual adjustments help listeners recognize reduced forms
Source: Front Psychol. 2014 May 30;5:437. doi: 10.3389/fpsyg.2014.00437 (PMC4038950; doi:10.3389/fpsyg.2014.00437)
Supplement: Supplementary file 1 [file DataSheet1.PDF]

Table A1. Target and competitor words in the segmental reduction condition of the test phase in Experiments 1, 2, and 3 with their word frequency per million according to SUBTLEX-NL (Keuleers, Brysbaert, & New, 2010).

| Test phase: segmental reduction condition | | | | | |
|---|---|---|---|---|---|
| /b/-target | English Translation | Word Frequency | /m/-competitor | English Translation | Word Frequency |
| banaal | banal | 1.2 | manuaal | manual | 0.0 |
| banaan | banana | 5.3 | mangaan | manganese | 0.1 |
| banier | banner | 0.5 | manie | mania | 0.5 |
| banket | banquet | 3.0 | mangrove | mangrove | 0.1 |
| bankier | banker | 3.7 | mankeren | to be wrong | 1.0 |
| bemachtigen | to get hold of | 3.3 | metallic | metallic | 0.2 |
| bemesten | to manure | 0.1 | memento | memento | 0.1 |
| bemoeien | to meddle | 12.1 | miljoenen | millions | 30.4 |
| benaming | name | 0.6 | mekaar | each other | 16.2 |
| benard | awkward | 0.0 | miljard | billion | 12.2 |
| benauwen | to oppress | 0.3 | mevrouwen | women | 0.1 |
| beneden | below | 188.0 | meneer | gentleman | 518.4 |
| benedictijn | Benedictine | 0.0 | menigvuldig | manifold | 0.0 |
| benedijen | to bless | 0.0 | menageren | to moderate | 0.0 |
| benoemen | to appoint | 2.5 | meloenen | melons | 1.6 |
| benoorden | north of | 0.0 | mesjokke | crazy | 0.4 |
| benutten | to utilize | 1.6 | mejuffrouw | Miss | 2.2 |
| benzine | gas | 24.7 | menslievend | charitable | 0.2 |
| binair | binary | 0.3 | mineur | minor | 0.4 |
| binderij | bookbinder | 0.0 | minderjarig | underage | 2.9 |
| binnenkort | soon | 45.5 | minnenswaard | lovable | 0.0 |
| binomisch | binomial | 0.0 | minoriteit | minority | 0.0 |
| bonbon | chocolate | 0.3 | mondain | faishonable | 0.2 |
| bonjour | good day | 2.6 | montuur | frame | 0.4 |
| Average | | 12.3 | Average | | 24.5 |
