# Supplementary material for: Use what you can: storage, abstraction processes, and perceptual adjustments help listeners recognize reduced forms
Source: Front Psychol. 2014 May 30;5:437. doi: 10.3389/fpsyg.2014.00437 (PMC4038950; doi:10.3389/fpsyg.2014.00437)
Supplement: Supplementary file 2 [file DataSheet2.PDF]

Table A2. Target and competitor words in the syllabic reduction condition of the test phase in Experiments 1, 2, and 3 with their word frequency per million according to SUBTLEX-NL (Keuleers, et al., 2010).

Test phase: syllabic reduction condition					
CVC-target	English Translation	Word Frequency	CC-competitor	English Translation	Word Frequency
charisma	charisma	0.8	chrisma	chrism	0.0
correct	correct	15.3	krek	just	0.1
coulisse	wing	0.0	klissen	to get entangled	0.1
curator	guardian	1.1	krater	crater	2.1
galei	galley	0.5	glei	thatch	0.0
galop	gallop	0.5	glos	gloss	0.0
genoom	genome	0.3	gnoom	gnome	0.3
gering	small	1.0	grind	gravel	0.7
kaneel	cinnamon	1.5	kneep	pinch	2.3
kanon	gun	6.2	knol	tuber	0.9
karaat	carat	1.1	kraak	crack	2.0
karaf	carafe	0.2	kras	scratch	2.1
karos	coach	0.1	krols	on heat	0.0
koliek	colic	0.2	kliek	clique	1.0
kolom	column	0.9	klomp	clog	0.9
kolonie	colony	6.4	clonen	to clone	0.1
kolos	colossus	0.4	klos	bobbin	3.4
konijn	rabbit	18.9	knijp	stuck	4.7
koran	Koran	2.0	kraan	tap	6.4
paraat	ready	6.2	praat	talk	241.8
Parijs	Paris	55.4	prijs	price	86.6
piloot	pilot	30.1	plooi	fold	0.6
polijsten	to polish	0.4	pleister	plaster	2.4
puree	puree	4.5	pree	pocket money	0.0
Average		6.4	Average		14.9
