# Supplementary material for: Use what you can: storage, abstraction processes, and perceptual adjustments help listeners recognize reduced forms
Source: Front Psychol. 2014 May 30;5:437. doi: 10.3389/fpsyg.2014.00437 (PMC4038950; doi:10.3389/fpsyg.2014.00437)
Supplement: Supplementary file 3 [file DataSheet3.PDF]

Table A3. Target words in the segmental and syllabic reduction condition of the exposure phase in Experiment 3 with their word frequency per million according to SUBTLEX-NL (Keuleers, et al., 2010).

Exposure phase: potentially reduced target words					
/b/-target	English Translation	Word Frequency	CVC-target	English Translation	Word Frequency
bandiet	bandit	4.7	caleidoscoop	kaleidoscope	0.2
bemanning	crew	20.4	canard	newspaper hoax	0.1
bemantelen	to cloak	0.0	Canossa	Canossa	0.0
bemerken	to notice	0.2	cholerisch	choleric	0.0
bemeten	sized	0.0	collaps	collapse	0.0
bemiddeling	mediation	0.2	college	lecture	13.3
beminnen	to love	1.8	collisie	collision	0.0
bemoederen	to mother	0.2	courage	courage	0.3
bemoedigen	to encourage	0.2	courant	newspaper	0.2
bemorsen	to soil	0.0	coureur	race-driver	2.0
bemost	bemoost	0.0	galactisch	galactic	0.2
benadelen	to harm	0.2	garantie	guarantee	6.1
benadering	approach	2.8	gareel	harness	1.2
benadrukken	to emphasize	2.2	generisch	generic	0.0
Benelux	Benelux	0.0	kanaal	channel	13.9
benemen	to take away	0.2	kinine	quinine	0.5
benepen	small-minded	0.2	kolere	cholera	6.6
benevens	besides	0.0	koraalrif	coral reef	0.2
benieuwen	to arouse curiosity	0.0	koraalrood	coral red	0.0
benijden	to envy	1.1	paleis	palace	15.5
benodigd	necessary	0.3	parochie	parish	2.5
benul	notion	2.9	piraat	pirate	6.4
bombardement	bombardment	1.8	polemisch	polemic	0.0
bombastisch	bombastic	0.0	politie	police	346.2
Average		1.6			17.3
