# Supplementary material for: Use what you can: storage, abstraction processes, and perceptual adjustments help listeners recognize reduced forms
Source: Front Psychol. 2014 May 30;5:437. doi: 10.3389/fpsyg.2014.00437 (PMC4038950; doi:10.3389/fpsyg.2014.00437)
Supplement: Supplementary file 4 [file DataSheet4.PDF]

Supplementary materials

Supplementary materials to "Use what you can: Storage, abstraction processes and perceptual adjustments help listeners recognize reduced forms" by Poellmann, Mitterer, and McQueen (2014).

This file contains the Dutch sentences used in the eye-tracking experiments. Underlined are the words that were reduced. In bold typeface appear the words that participants should click on.

1. Test sentences of Experiments 1 - 3

1.1. Reduced /b/-words

Ik heb alleen het eerste deel van je e-mail tot **banaal** ontvangen.
In sommige talen lijken woorden zoals **banaan** erg op elkaar.
Voor een referaat moet ik meer over de **banier** te weten komen.
Je zal nuttige documenten vinden, als je bij google **banket** intypt.
In de Franse les moest ik een zin met **bankier** formuleren.
Op deze plaats werd **bemachtigen** verkeerd vertaald.
Natuurlijk wist hij het wachtwoord **bemesten** uit zijn hoofd.
Zij moest het twee keer opnieuw vragen totdat ze eindelijk **bemoeien** verstond.
Hij spraak met zo 'n hoge stem dat **benaming** heel vreemd klonk.
Vanwege het rumoer in de achtergrond kon hij **benard** nauwelijks verstaan.
De scholieren moesten een ander woord voor **benauwen** vinden.
Omdat hij zo slecht hoorde, moest zij **beneden** drie keer herhalen.
Omdat het inktpatroon leeg was, kon hij **benedictijn** niet meer goed lezen.
In een examen moest hij **benedijen** met zijn eigen woorden omschrijven.
Omdat hij stotterte, kreeg hij het woordje **benoemen** er met moeite uit.
In de e-mail was **benoorden** verkeerd geschreven.
Omdat Nederlands niet haar moedertaal was, verstond zij **benutten** niet meteen.
Hij had niet opgelet en wist daarom het antwoord **benzine** niet.
Voor de geheime ontmoeting kreeg alleen diegene toegang, die het woord **binair** kende.
Het tekstverwerkingprogramma kende het woordje **binderij** niet.
De auteur wilde de herhaling van **binnenkort** vermijden.
Om eerst een algemeen overzicht te krijgen, kun je bij wikipedia naar **binomisch** zoeken.
Omdat hij zo zacht praatte, was **bonbon** nauwelijks te horen.
In een invuloefening moesten de kinderen **bonjour** invoegen.

1.2. Canonical /m/-words

Het winnende woord van de dag was **magazijn**.
Het was makkelijker dan ze had gedacht om een **majoraan** te tekenen.
Het team bracht met brainstormen over **makelij** een opmerkelijk resultaat tot stand.
Bij nader toezien ontdekte zij een **Maleis** op de foto.
Het was wel grappig, maar een hit werd het lied over een **malloot** niet.
Het meisje kleurde met overgave een **marron** in een kleurboek.
In een vaktijdschrift kwam heel vaak het woordje **masculien** voor.

Echt veel punten krijg je bij scrabble voor het woordje **massaal** niet.
Je kunt een leuke prijs winnen, als je de oplossing **masseren** inzendt.
Dat krantenartikel over het **massief** was vrij zorgwekkend.
Een moeilijke quizvraag ging over de definitie van **mastiek**.
Trots liet hij zijn vrienden een foto van een **matador** zien.
Deze keer zal zijn lezing over **migratie** gaan.
Hij stuurde een sms met het codewoord **milieu** naar 4433.
Ik had niet gedacht dat een artikel over de **mistral** zo interessant zou zijn.
Op de veiling werd het schilderij van een **moeras** voor 400€ verkocht.
Er zijn mensen die bij zichzelf **moreel** op de rug laten tatoeëren.
Vanaf deze afstand kon zij het woord **motorisch** niet meer lezen.
Bij de basiswoordenschat horen woorden zoals **mousseren** zeker niet.
De schilder vond snel een koper voor zijn **mouterij** in olieverf.
Het is vrij makkelijk om **moveren** van achteren naar voren te spellen.
Voor Duitsers is het moeilijk om **muiterij** juist uit te spreken.
In een woordenboek zul je zeker de definitie van **mulattin** vinden.
Er stonden slechts een paar woorden zoals **mutant** op het briefje.

1.3. Reduced CVC-words
Het enige dat ze kon ontcijferen was **charisma**.
Bij vergissing vertaalde de tolk het woord **correct** niet.
In een rijmwoordenboek staan zeker ook woorden die op **coulisse** rijmen.
De lerares legde aan de kinderen uit wat een **curator** was.
Zij vond een stukje papier waarop alleen **galei** geschreven stond.
Hij heeft zich versproken en **galop** gezegd.
Hoewel zij zich echt inspande, kon ze slechts **genoom** opvangen.
Omdat zij niet goed oplette, hoorde zij het woord **gering** niet.
Voor Duits moest de scholier een woordenlijst leren, waar ook **kaneel** op stond.
De cursisten moesten een toneelstuk improviseren, waarin **kanon** voorkwam.
Hij moest voor zijn medespelers **karaat** omschrijven.
Hoewel hij nauwlettend zocht, kon hij geen **karaf** ontdekken.
In het kruiswoordraadsel werd naar een ander woord voor **karos** gevraagd.
Zonder enige aanleiding heeft hij plotseling **koliek** gezegd.
Ten slotte vond hij de **kolom** die hij zocht.
Hij kon zich niet meer herinneren hoe men **kolonie** spelde.
Toen zij haar ogen opende zag zij daar een **kolos** staan.
Totaal zonder aanleiding kwam ineens **konijn** in haar op.
Naar lang zoeken zag hij er een **koran** liggen.
Wat hij ook zei, hij had zeker **paraat** voor ogen.
Ze wist alleen nog dat **Parijs** in de titel voorkwam.
Met dit volume kon hij alleen **piloot** verstaan.
De vertaler wist het Franse woord voor **polijsten** niet.
Dat is zeker een typefout en moet **puree** zijn.

1.4. Canonical CC-words
Het Spaanse equivalent voor **glans** wist hij niet meer.

Omdat zijn ogen zo slecht waren, kon hij **grazen** nauwelijks lezen.
Het was makkelijk om met pantomime het woord **grillen** uit te beelden.
Van de laatste les stond nog steeds **klager** op het bord.
Omdat het in braille geschreven was, kon ze **kleden** zelfs in het donker lezen.
We meten ook jouw reactietijd als je **klemtoon** hoort.
Omdat het dikgedrukt was, sprong **kletsen** meteen in het oog.
Hij had het foute antwoord gekozen toen hij voor **klieven** koos.
Voor een eersteklasser is **klipper** een lastig woord.
De laatste keer moest hij bij het spel hints **klontje** representeren.
In de tekenles moesten de kinderen een **knaagdier** schilderen.
Hoewel het raadsel echt moeilijk was, heeft hij de oplossing **knersen** gevonden.
Het viel hem tegen om het woord **kraagje** juist te schrijven.
Zonder haar bril kon ze het woordje **krachtig** niet scherp zien.
Op het schilderij was een prachtige **kriek** getekend.
Het laatste woord dat nog op de bladzijde paste, was **kronen**.
In dit experiment staan woorden zoals **krullen** op het scherm.
Soms moeten proefpersonen vaststellen of **plaag** een Nederlands woord is.
Op een kladblaadje stond alleen **plek** geschreven.
In een heel moeilijk dictee kwam het woord **prangen** voor.
Je moet nu snel op het woordje **prater** klikken.
De dichter kon geen rijmwoord voor **preuts** vinden.
Ze wist niet precies, waar **pront** voor stond.
Omdat ze dyslectisch was, kon ze **prooi** niet vlot voorlezen.

2. Experiment 1: Exposure sentences

2.1. (Potentially) Reduced /b/-words
Haar opmerking was echt <u>banaal</u> en **nietszeggend**.
Ik wil nu geen <u>banaan</u>, maar wel een **appel**!
Een <u>banier</u> is een vlag die het **wapen** van een adellijke familie toont.
Het <u>banket</u> ter ere van de koningin was een doorslaand **succes**.
Een <u>bankier</u> van een Zwitsers instituut heeft onlangs twee miljard **dollar** fout geïnvesteerd.
Ik heb daadwerkelijk kaartjes kunnen <u>bemachtigen</u> voor het **concert** van Guus Meeuwis!
Als boeren hun velden <u>bemesten</u>, kun je dat in de hele buurt **ruiken**.
Mensen die zich overal mee <u>bemoeien</u>, zijn heel **lastig**.
Meestal is het heel moeilijk een simpele <u>benaming</u> voor een complexe **zaak** te vinden.
De situatie was erg <u>benard</u> en ik wilde alleen nog maar **weg**!
Al die verplichtingen <u>benauwen</u> me zo, dat ik vaak niet goed kan **slapen**.
Met temperaturen <u>beneden</u> de min 20 was het een heel koude **winter**.
De <u>benedictijn</u> Dom Pérignon heeft de productie van **champagne** enorm verbeterd.
<u>Benedijen</u> is een oud woord voor **zegenen**.
Het <u>benoemen</u> van een professor is altijd een **langdurig** proces.
Waarschijnlijk was de schat ooit <u>benoorden</u> de hut **verborgen**.
Huntelaar wist de strafschop te <u>benutten</u> en bracht zo zijn team aan de **leiding**.

In de eerste week van de vakantie stijgt de vraag naar benzine altijd **enorm**.
Computers rekenen in een binair stelsel, waarvan een **bit** de kleinste eenheid is.
Pas in een binderij wordt een boek of **tijdschrift** afgemaakt.
Ik verwacht binnenkort een **pakje** van Amazon.
Als ik binomisch hoor, krijg ik meteen nachtmerries over **wiskunde**.
Die lekkere bonbon heb ik in een Franse **chocoladewinkel** gekocht.
Als je in Frankrijk bent, moet je bonjour in plaats van **dag** zeggen.

2.2. Canonical /m/-words

We kunnen niet door de tijd reizen, omdat nog niemand een dergelijke **machine** heeft uitgevonden.
Harry Potter moest naar Zweinstein gaan om **magie** te leren gebruiken.
Het kwam tot een echtelijke ruzie toen de vrouw over de **maîtresse** van haar man te weten kwam.
Er zou bijna een ongeluk zijn gebeurd, toen de Mercedes de vrachtwagen bij een gevaarlijke **manoeuvre** inhaalde.
De auteur wist al naar welke uitgeverij hij zijn **manuscript** wilde sturen.
Voor een Duitse rug is een Nederlands **matras** vaak veel te zacht.
Hoewel hij niet op een schip werkt, is Popeye altijd als een **matroos** gekleed.
Het orkest speelde een prachtige **melodie** ter afsluiting van het concert.
Hij kon zich fysiek niet met zijn vrienden meten, maar **mentaal** was hij hen de baas.
Naar dit eetcafé gaan is altijd een nieuwe ervaring, omdat de **menukaart** zo vaak wisselt.
Ik zou het gedrag van die vent niet normaal noemen, maar **merkwaardig** wel!
Voor de Nederlandse krijgsmacht kun je als vrouw sinds 1951 als **militair** in dienst gaan.
Ik hield van films met Charlie Chaplin vanwege de grappige **mimiek** die hij heeft!
Met een aandeel van 12% in de aardkorst is kwarts het meest voorkomende **mineraal** op aarde.
Spiegelreflexcameras zijn vrij duur, dus je moet met een prijs van **minimaal** 500€ rekening houden.
Ik heb net een ipad gekocht, en nog wel het nieuwste **model** op de markt!
Vroeger was het veilig om naar Japan te reizen, maar **momenteel** is dat niet het geval.
De staatsvorm van Nederland is een constitutionele **monarchie**.
De toehoorders vielen bijna in slaap, omdat de man zo **monotoon** sprak.
Mijn computer is al dagen kapot, maar vandaag is de **monteur** eindelijk langs gekomen.
Veel oude gebouwen zoals de Dom van Utrecht zijn als **monument** erkend.
De terrorist doodde zichzelf toen hij bijna zijn hele **munitie** had verschoten.
Soms worden ook werken van Picasso in het Van Gogh **museum** tentoongesteld.
D'Artagnan wilde net als zijn vrienden **musketier** worden.

2.3. (Potentially) Reduced CVC-words

Mensen met charisma kunnen gemakkelijk **invloed** op anderen uitoefenen.
Het antwoord was niet helemaal correct, waardoor ik niet de hoogste **waardering** kreeg.
Als coulisse wordt in een openluchttheater gewoon de **natuur** gebruikt.
Als je failliet bent, krijg je van de rechter een curator, een soort **voogd,** toegewezen.
Als je dienst moest doen op een galei, was dat vaak een **doodvonnis** in het Oude Rome.
In gestrekte galop reed hij over de **eindstreep**.

Pas in 2000 was het mogelijk het menselijke genoom te **ontrafelen**.
Hun leeftijdsverschil is dan wel niet gering, maar ze kunnen toch goed met elkaar **opschieten**.
De geur van kaneel is typisch voor de **kersttijd**.
Hij was zo zat als een kanon, maar hij wilde per se alleen naar huis **fietsen**.
Een vriendin van mij heeft een ring van 18 karaat goud voor haar **verjaardag** gekregen!
Bij een diner wordt een karaf water niet altijd **gratis** geserveerd.
Een karos is een prachtige **koets** met vier wielen.
Bij een koliek heeft een **paard** onder andere ernstige buikpijn.
Meestal staat pas in de laatste kolom de echt interessante **informatie**.
De Nederlandse kolonie Suriname werd in 1975 **onafhankelijk**.
Toen ik de vorige keer aan het joggen was, stond er opeens een kolos van een **hond** voor mij.
Veel kinderen willen graag een schattig konijn als **huisdier**.
Je moet de koran kunnen lezen, als je tot de **islam** wilt overgaan.
Normaalgesproken heeft hij altijd een antwoord paraat, maar deze keer wist hij niets meer te **zeggen**.
De Eiffeltoren staat in Parijs aan de linkeroever van de **rivier** de Seine.
Vanwege een probleem met de motor moest de piloot een **noodlanding** maken.
Ik moet eerst nog mijn nagels polijsten, voordat ik kan **uitgaan**.
Duitsers houden van aardappelen in allerlei vormen, onder andere als puree, gebakken en uit de **oven**.

2.4. Canonical CC-words
Als het toilet pas schoongemaakt is, kun je de geur van **chloor** vaak nog ruiken.
PSV Eindhoven is op dit ogenblik een succesvolle **club** in de eredivisie.
Vaak snap ik poëzie niet echt, omdat het zo **cryptisch** kan zijn.
Voor persoonlijk gebruik mag je maximaal 5 **gram** wiet bij je hebben.
Als je op het gymnasium zit, moet je Latijn en **Grieks** leren.
In de winter hebben veel mensen **griep** onder de leden.
Het water van het vijver zag er **groenachtig** uit.
Een succesvolle reclame moet op de **klantenkring** afgestemd zijn.
Mijn zus en haar nieuwe vriend zijn pas bij elkaar en zitten de hele tijd **klef** te doen.
Prinses Eloise is de oudste **kleindochter** van Beatrix.
Aan dit experiment mag je ook deelnemen als je **kleurenblind** bent.
Traditionele middelen tegen vampiers zijn **knoflook** en wijwater.
Om een ongeluk af te weren, klopt men met de **knokkel** op een stuk hout.
Als Ajax de voorsprong van 3-0 nog uit handen geeft, zou dat erg **knullig** zijn.
Op de marktdag biedt iedere **kramer** zijn koopwaar aan.
Een soldaat die opzettelijk burgers doodt, moet dat voor een **krijgsraad** rechtvaardigen.
In plaats van koffie kun je voor ontspanning beter **kruidenthee** drinken.
Een muilezel ontstaat bij een **kruising** tussen een paard en een ezel.
Op een bouwplaats mag een **kruiwagen** niet ontbreken.
Voor veel walvissoorten is **plankton** het voornaamste voedsel.
Als je in een formele situatie geen standaard Nederlands, maar **plat** praat, geeft dat geen goede indruk.

Bij sollicitaties wordt ervaring in het buitenland als **pluspunt** gezien.
Een secretaresse zou zonder telefoon, computer en **printer** nauwelijks kunnen werken.
Ook bij de volgende uitreiking van de Oscars zullen de sterren weer met hun kleren **pronken**.

3. Experiment 2: Exposure sentences

3.1. (Potentially) Reduced /b/-words

Als in wiskunde een samenhang triviaal is, moet de redenering ook echt banaal zijn.
Niet alleen mensen, maar ook apen eten graag een banaan.
Een vlag die het wapen van een adellijke familie toont, noemt men een banier.
Het heerlijke gebak voor haar verjaardag kwam van de beste banketbakker uit de stad.
Na de redding van Fortis waren vele mensen kwaad over de bonussen van de bankiers.
Voor het concert van Guus Meeuwis heb ik daadwerkelijk kaartjes kunnen bemachtigen!
Je kunt het in de hele buurt ruiken als boeren hun velden bemesten.
Mensen kunnen heel lastig zijn als ze zich overal mee bemoeien.
Voor sommige uitvindingen is het heel moeilijk een treffende benaming te vinden.
Ik voelde me erg angstig, want de situatie was erg benard!
In de zomer wordt het op zolder erg benauwd.
Mijn rapportcijfer van 7,4 werd afgerond naar beneden.
Aan de pij kun je zien of een monnik augustijn, franciscaan of benedictijn is.
In plaats van "zegenen" komt in het Heilige Schrift soms ook "benedijen" voor.
Het is een langdurig proces, als een universiteit een nieuwe professor moet benoemen.
"Ten noorden van" wordt veel vaker gebruikt dan "benoorden".
Huntelaar bracht zijn team aan de leiding door een strafschop te benutten.
Mijn Opel heeft geen diesel, maar gewone benzine nodig.
Een getalstelsel waarin uitsluitend de waarden 0 en 1 voorkomen, is een binair stelsel.
Als een manuscript gedrukt is, moet het naar de binderij.
Ik wacht op een pakje van Amazon dat hopelijk binnenkort verstuurd wordt.
Ik krijg meteen nachtmerries over wiskunde, als ik "binomisch" hoor.
Een Franse chocoladewinkel had deze lekkere bonbon verkocht.
In plaats van "goedendag" moet je in Frankrijk "bonjour" zeggen.

3.2. Canonical /m/-words

Same as in Experiment 1 (see 2.2.)

3.3. (Potentially) Reduced CVC-words

De sterke persoonlijke aantrekkingskracht die iemand uitoefent op andere mensen noemt men charisma.
Ik kreeg niet de hoogste waardering, omdat mijn antwoord niet helemaal correct was.
Na een optreden kleedden de toneelspelers zich om achter de coulissen.
De voorbereiding van een tentoonstelling en de oprichting van een verzameling horen tot de taken van een curator.
In het Oude Rome was het vaak een doodvonnis, als je als slaaf moest roeien op een galei.
Eerst liet zij het paard draven, toen bracht ze het in galop.

Alle erfelijke factoren tezamen die in een individu aanwezig zijn, vormen het genoom.
Bij een ruggenprik is de kans op een infectie zeer gering.
Appeltaart maak je vaak met appels, rozijnen en wat specerijen, waaronder kaneel.
Een gewichtig wapen voor een zeeslag was tot in de Tweede Wereldoorlog het kanon.
Voor haar verloving heeft Suzanne een ring van 18 karaat goud gekregen.
Bij een diner wordt wijn vaak in een karaf geserveerd.
Een prachtige koets met vier wielen noemt men ook een karos.
Als een paard erge pijn in zijn buik heeft, heeft het waarschijnlijk een koliek.
De duur van alle prikkels vind je in deze tabel in de derde kolom.
Tot 1975 was Suriname een Nederlandse kolonie.
Aan het enorme wereldwonder van Rodos hield de Nederlandse taal het woord kolos over.
Als huisdier willen veel kinderen graag een schattig konijn.
Als je tot de islam wil overgaan, moet je het Heilige Schrift, de koran, kunnen lezen.
Normaalgesproken heeft hij altijd een antwoord paraat.
Disneyland, de Eiffeltoren en het Louvre lokken elk jaar miljoenen mensen naar Parijs.
In de film "The Aviator" speelt Leonardo DiCaprio een succesvol ondernemer en piloot.
Om extra schone tanden te krijgen, wil ik ze door de tandarts laten polijsten.
Duitsers houden van aardappelen in allerlei vormen, zoals gebakken of als puree.

3.4. Canonical CC-words

Same as in Experiment 1 (see 2.4.)

4. Experiment 3: Exposure sentences

4.1. (Potentially) Reduced /b/-words

In Lucky Luke is Joe Dalton de kleinste en slimste **bandiet** van de vier Dalton broers.
Toen het vliegtuig neerstortte, is de hele **bemanning** om het leven gekomen.
Omdat het schandaal zijn carrière zou ruïneren, deed hij zijn best om het te **bemantelen**.
Ze was heel zenuwachtig, maar godzijdank scheen niemand haar plankenkoorts te **bemerken**.
Ik heb pas net op tijd mijn werk af; de termijn was echt krap **bemeten**.
Als je een huis wilt kopen, ben je aangewezen op de **bemiddeling** van een makelaar.
Met Julia Roberts in de hoofdrol werd de bestseller "Eten, Bidden, **Beminnen**" onlangs verfilmd.
Sommige ouders kunnen het instinct hun kind te **bemoederen** nauwelijks inhouden.
Met zijn glimlach en zijn vriendelijke woorden wilde hij haar **bemoedigen**.
Zij had nog maar net schone kleren aan, toen zij het voor elkaar kreeg om zich weer te **bemorsen**.
Vaak zie je dat de bomen aan één kant heel groen en dus erg **bemost** zijn.
Als je zoveel blijft roken, zul je niet alleen jouw gezondheid maar ook de mijne **benadelen**.
De baas was het niet met hem eens over de oplossing van het probleem en keurde zijn **benadering** niet goed.
We snapten het de eerste keer al, je hoeft het dus niet zo te **benadrukken**.
België, Nederland en Luxemburg werken samen als de **Benelux** unie.

Zij was zo wanhopig dat ze geen andere uitweg zag dan zich van het leven te **benemen**.
Toen hij de bal tegen de vaas schopte, kreeg de jongen een **benepen** gezicht.
Voor deze baan zou hij 3000€ per maand **benevens** dienstauto en iphone krijgen.
Omdat hij altijd te laat komt, zal het me **benieuwen** wanneer hij precies opdaagt.
Zij is nooit tevreden met dat wat ze heeft en blijft andere mensen **benijden**.
Om brood te bakken is gist een **benodigd** ingrediënt.
Hij doet alsof hij alles weet, maar in werkelijkheid heeft hij er geen flauw **benul** van.
In 1944 werd Nijmegen per ongeluk door een Amerikaans **bombardement** verwoest.
Het optreden van de kunstenares was heel theatraal en **bombastisch**.

4.2. Canonical /m/-words
Same as in Experiment 1 (see 2.2.)

4.3. (Potentially) Reduced CVC-words
Kinderen spelen graag met een **caleidoscoop**, omdat ze van de mooie kleuren en vormen houden.
Op 1 april blijken sommige artikelen een **canard** te zijn.
Als iemand publiekelijk boete moet doen, noemt men dat ook wel "de gang naar **Canossa** maken".
Iemand die snel driftig wordt, heeft een **cholerisch** temperament.
Een oude dame dronk met deze hitte niet voldoende water en heeft een **collaps** gekregen.
Omdat de professor ziek is, kan hij vandaag geen **college** geven.
De oorzaak van de file was een **collisie** van een vrachtwagen en een auto.
Om ridder te worden, moest een man veel **courage** hebben.
Voor nieuws uit Friesland kun je het best de Leeuwarder **Courant** lezen.
Sebastian Vettel is momenteel de beste **coureur** van de wereld.
Met een telescoop zie je in het sterrenbeeld Orion ook een **galactisch** stelsel.
Ik heb op mijn nieuwe fototoestel drie jaar **garantie** gekregen.
De delinquente jongeren moesten in het internaat in het **gareel** gehouden worden.
Een kopie van een merkmedicijn noemt men ook een **generisch** medicijn.
De Oudegracht is het mooiste **kanaal** in Utrecht.
Tegen malaria was voor de ontdekking van het medicijn Lariam alleen **kinine** verkrijgbaar.
Ik zeg niet vaak "Krijg de **kolere**!", alleen als ik echt woedend ben.
De duikers wilden in Australië hoofdzakelijk het grote **koraalrif** zien.
Mijn dochter wil eruitzien als Ariël de zeemeermin en haar haar **koraalrood** verven.
Tijdens een verblijf in Amsterdam moet je ook het koninklijk **paleis** eens zien.
Om nieuwe aanhangers te winnen, is preken voor eigen **parochie** niet echt van groot nut.
Voor carnaval heb ik me dit jaar als **piraat** verkleed.
De journalist vond het leuk om **polemisch** te schrijven.
Kort na de bankoverval kon de **politie** de dader arresteren.

4.4. Canonical CC-words
Same as in Experiment 1 (see 2.4.)
